# Spreadable Biosensing Pregel for Analyte Visualization in Peeled Plant Tissues

**DOI:** 10.1021/acs.analchem.6c01240

**Published:** 2026-07-06

**Authors:** Hayelom Dargo Beyene, Decibel P. Elpa, Pawel L. Urban

**Affiliations:** Department of Chemistry, 34881 National Tsing Hua University, 101, Section 2, Kuang-Fu Rd., Hsinchu 300044, Taiwan

## Abstract

Biosensors are typically fabricated in the form of solid-state devices (chips, tapes, or films). Here, we demonstrate a proof-of-concept for an alternative form of biosensor—an amorphous biosensing pregel, which is formed on the interrogated surface of interest after application of a precursor mixture. The reagent cocktail is prepared from dilute agarose and a mixture of assay chemicals, including enzymes and substrates. The readout is either colorimetric or luminometric, and it is facilitated by a digital camera. The main conditions of the biosensing pregel preparation and application have been optimized. The method is particularly suitable for rudimentary macroscopic visualization of metabolites in plant tissues. In one exemplary application, plant leaves are split into two sides using adhesive tape. Following the application of the biosensing cocktail, distributions of target species such as glucose and adenosine triphosphate can be visualized in the peeled tissues. Thus, the approach enables rapid in situ sensing of analytes in the studied flat specimens without fabrication and use of classical solid-state biosensors.

## Graphical abstract

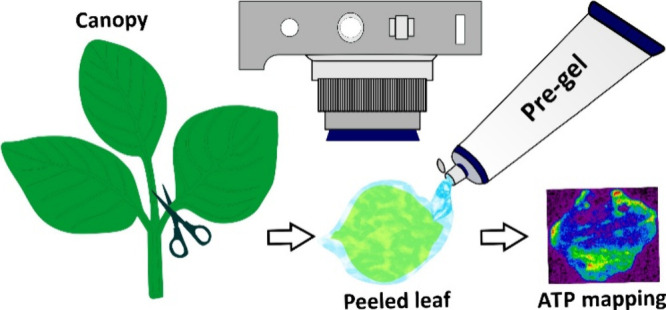

## Introduction

Single-point and imaging biosensors are tools that detect analytes based on biochemical reactions, each offering distinct advantages. Specifically, single-point biosensors for colorimetric, electrochemical, and bioluminometric detection of glucose,^1–4^ ascorbic acid (AA),^5–7^ and adenosine triphosphate (ATP)^8,9^ have been reported. They generally enable rapid measurements and are characterized with straightforward operation. However, they are limited in their ability to provide spatial information about analyte distribution, which is crucial for understanding molecular interactions and chemical heterogeneity within tissues. In contrast, imaging biosensors capture entire sample images, enabling detailed spatial analysis.^10^ For instance, Jin and co-workers showed that common face coverings can capture and map aerosolized saliva droplets, using colorimetric biosensing strips in smart masks to detect the salivary biomarker α-amylase across four mask types.^11^ Bioluminescence imaging has enabled the spatial mapping of key energy metabolites—such as ATP, glucose, glycogen, and lactate—within frozen pig and rabbit arteries^12,13^ Other works focused on mapping of sucrose in*Vicia faba* cotyledons^14^ and hydrogen sulfide (H_2_S) in cancer cells and mouse tissues.^15^

Biosensors provide rapid, sensitive, and noninvasive detection of biochemical analytes and have become effective tools for monitoring plant physiological states.^16^ Electrochemical biosensors enable nondestructive, in situ detection of biochemical species.^17^ Plant biosensor platforms have been developed for glucose,^16–18^ and ATP sensing,^9^ offering insights into plant stress responses and metabolic regulation. These approaches have been successfully used to track leaf-level stress responses,^16^ investigate sugar homeostasis in tree vascular tissues,^19^ and monitor extracellular ATP transport under physiological and stress conditions.^9^ In addition, plant biosensors have been engineered to detect key dissolved phytohormones, including abscisic acid,^20^ salicylic acid,^21^ and indole-3-acetic acid,^22^ which regulate plant growth, development, and environmental responses. Despite these advances, most existing methods are single-point biosensors and cannot resolve the spatial heterogeneity of metabolites across tissue surfaces.^9,23^ Other electrochemical detection systems are invasive, depend on complex sensor arrays, and are difficult to deploy in resource-limited environments.^17,18,21,24,25^ Colorimetric and luminometric approaches for spatial mapping of analytes in plant tissues remain underexplored. Therefore, here we demonstrate spreadable biosensing pregels that can be directly applied to plant tissue surfaces for analyte visualization. They can be used for mapping glucose, ATP, and AA on surfaces of plant tissues.

Hydrogels have emerged as promising materials for biosensor applications due to their tailorable properties,^26^ biocompatibility,^27^ and sensitivity to external stimuli.^28^ These characteristics make hydrogels suitable for a wide range of biosensing applications, from detecting biomolecules to monitoring environmental changes.^29^ Li and co-workers visualized rhizosphere pH dynamics using agar gel with bromocresol purple, demonstrating that the fava bean rapidly acidifies the rhizosphere—thereby increasing phosphorus solubility and facilitating phosphorus uptake in maize—while maize alkalizes the rhizosphere.^30^ Hydrogel-based biosensors employ various signal transduction methods, including surface plasmon resonance,^31^ and electrochemiluminescence,^32^ enabling rapid response, excellent stability, and reproducibility in detecting a wide range of biological targets. Various types of hydrogels have been developed for biosensing applications, including conductive hydrogels.^33,34^ smart hydrogels,^35^ and nanofiber hydrogels.^36^ Conductive hydrogels, which combine the mechanical properties of hydrogels with electrical conductivity, are particularly promising for applications in wearable sensors and bioelectronics.^34^ They exhibit tunable conductivity and self-healing properties, making them suitable for stretchable and flexible devices.^37^ Smart hydrogels, which respond to external stimuli such as pH, temperature, or light, can be engineered to change their properties in real-time, thus enhancing their functionality in biosensing applications.^38,39^

Recent advancements highlight the potential of hydrogels for immobilizing enzymes in biosensors.^40,41^ They demonstrate improved enzyme stability, reusability, and activity, and parallel the trend of deploying enzymatic microreactors in biotechnology.^42^ Hydrogels provide an optimal environment for trapping enzymes, enabling controlled release and substrate interaction.^43^ Various hydrogels have been developed for enzyme immobilization, including those based on biopolymers such as sodium alginate,^44,45^ chitosan,^46^ and poly­(vinyl alcohol).^47^ The physical and chemical properties of hydrogels can be tailored to optimize enzyme immobilization and substrate diffusion while retaining the enzyme within the matrix and enhancing catalytic efficiency by reducing the distance for substrate transfer.^48^ Enzyme immobilization in hydrogels improves enzyme activity and allows for the development of multienzyme systems that can catalyze complex reactions more effectively.^48^

The hydrogel-enzyme matrices used in biosensors range from soft to semisolid, often requiring nanoparticles as well as supporting platforms—such as films, micropatches, or arrays—to maintain structure and analyte contact. Here, we present a spreadable biosensing pregel format for enzyme and reagent delivery that is simple to prepare, easy to use, and compatible with both colorimetric and bioluminescence imaging. The system employs an amorphous spreadable biosensing pregel that can be directly applied to sample surfaces without additional supporting materials, enabling spatial mapping of analyte distributions. Unlike conventional hydrogel biosensors confined within rigid formats, this approach allows for direct interfacing with complex surfaces. Its utility is demonstrated through mapping of glucose and ATP in plant leaves and AA in citrus fruit cross sections. To our knowledge, the use of an unconfined, spreadable biosensing pregel for direct analyte mapping on peeled leaf surfaces has not been previously reported.

## Experimental Section

### Chemicals and Specimens

Agarose (ultra grade for molecular biology) was purchased from UniRegion Bio-Tech (New Taipei City, Taiwan). 4-Aminoantipyrine (4-AAP, 98%), 2,6-dichloroindophenol sodium salt hydrate (Tillman’s (TM) reagent), d-(+)-glucose (99.5%, GC grade), ethylenediaminetetraacetic acid (EDTA), magnesium sulfate, peroxidase from horseradish (HRP; E.C. 1.11.1.7, type II, cat. no. P8250), sucrose (99.5%), and tris­(hydroxymethyl)­aminomethane (Trizma base; crystalline) were purchased from Sigma-Aldrich (St. Louis, MO, USA). Phenol (99%) was purchased from Alfa Aesar (Ward Hill, MA, USA). Methyl red, sodium phosphate dibasic dihydrate (99%) and sodium phosphate monobasic (99%) were purchased from Acros Organics (Geel, Belgium). Glucose oxidase from *Aspergillus niger* (GOx; E.C. 1.1.3.4, cat. no. 195196) was purchased from MP Biomedicals (Santa Ana, CA, USA). AA was purchased from J.T. Baker (Phillipsburg, NJ, USA). Adenosine triphosphate (ATP) sodium salt was purchased from TCI (Tokyo, Japan). d-Luciferin potassium salt (in vivo grade, cat. no. P1043) and luciferase (E.C. 1.13.12.7, QuantiLum recombinant, cat. no. E1701) were purchased from Promega (Madison, WI, USA). Hydrochloric acid was purchased from Merck (Darmstadt, Germany). Toluidine Blue O (TBO) was purchased from Thermo Scientific (Waltham, MA, USA).

For glucose and ATP imaging, spearmint (*Mentha spicata*), parsley (*Petroselinum crispum*), and cilantro (*Coriandrum sativum*) plants were purchased from a local market (Hsinchu, Taiwan). For AA imaging, citrus fruits—kiwi (origin, Italy), lemon (origin, Taiwan), and orange (origin, USA)—were purchased from a local market (Hsinchu, Taiwan) and stored in the fridge (∼4 °C) before use. The fruits were sliced into ∼7 mm-thick sections, dried in a hot-air oven at 60 °C for 4 h, and then stored in a desiccator before analysis.

### Pregel Preparation for the Detection of Glucose

To prepare the biosensing cocktail with enzymes for glucose detection and visualization (Figure 1Ai–iii), the agarose-water mixture was heated in a water bath at ∼90 °C and stirred at 300 rpm until it formed a transparent solution. Once achieved, the agarose solution was cooled to ∼40 °C—below the thermal transition temperature of GOx and HRP.^49–51^ Next, the following reagents were added in sequence: GOx, 4-AAP, phenol, and HRP solution prepared in sodium phosphate dibasic dihydrate and sodium phosphate monobasic at pH 6. The final pregel cocktail contained 2 g L^–1^ agarose, 50 mM 4-AAP, 50 mM phenol, 5 U mL^–1^ GOx, and 5 U mL^–1^ HRP. For method development and testing, a glucose sample was prepared by pipetting 100 μL of 1 mM d-(+)-glucose onto the surface of a 10 mm diameter circle drawn on wax paper and allowed to dry. Then, a 200 μL aliquot of the biosensing cocktail was pipetted to detect glucose. For specimen analysis, a 0.1–1 mL aliquot of the cocktail was pipetted onto the surface.

**Figure 1:**
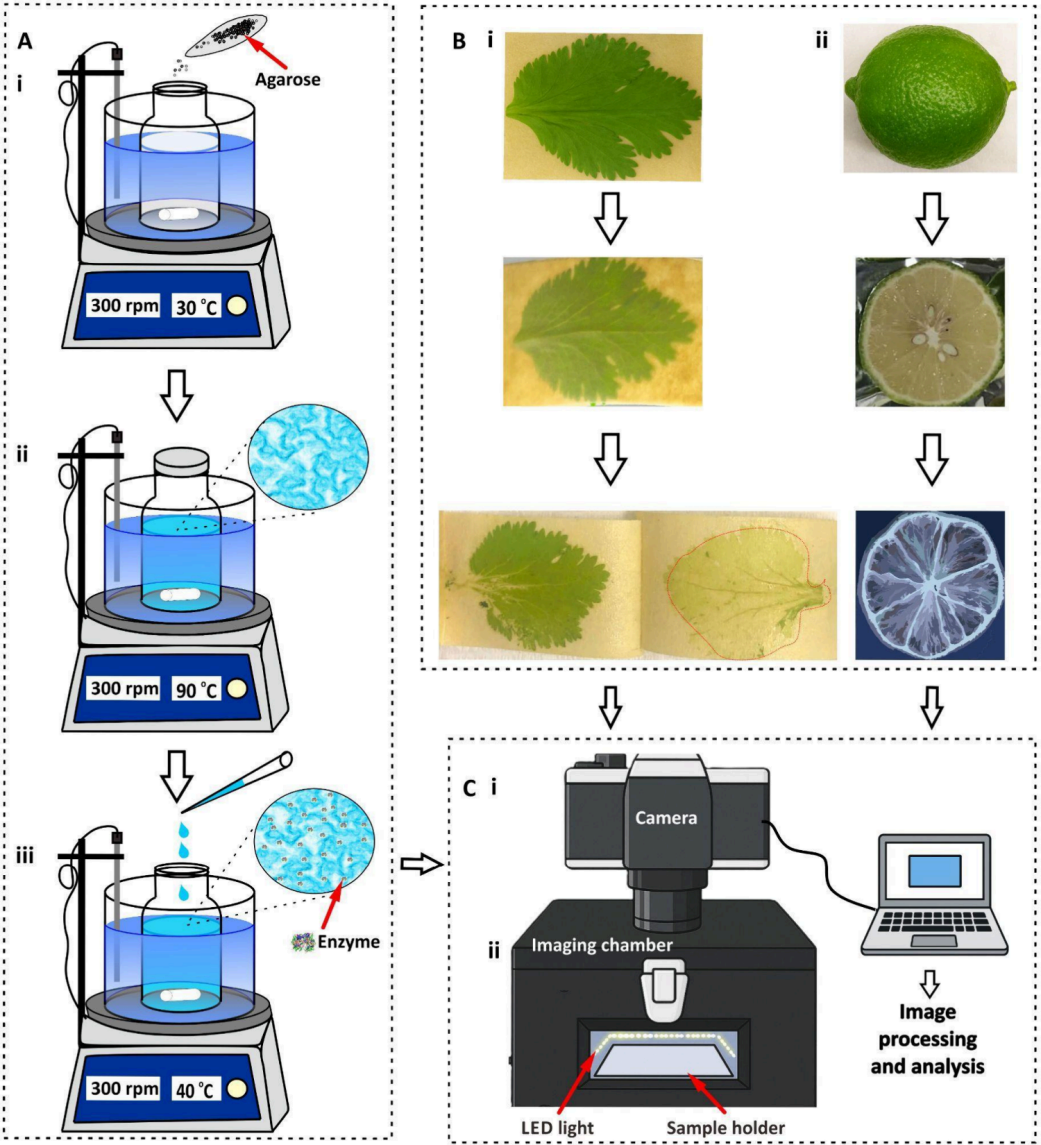
Workflow for analyte detection using the spreadable biosensing pregel. (A) Reagent cocktail preparation: (i) addition of agarose powder for subsequent dissolution in distilled water; (ii) heating and stirring the water–agarose mixture at 90 °C and 300 rpm until transparent; (iii) cooling the agarose pregel to 40 °C before adding enzymes and reagents. (B) Sample preparation: (i) separation of the lower leaf epidermis from the upper tissue using a tape-leaf sandwich; (ii) a section of sliced and dried fruit tissue. (C) Imaging system for capturing colorimetric and luminometric images: (i) digital camera connected with a laptop computer; (ii) imaging chamber where an LED light source provided illumination during image acquisition.

### Pregel Preparation for the Detection of ATP

To prepare the biosensing cocktail with enzymes for ATP detection (Figure 1A­(i,ii)), the agarose-water mixture was first heated to ∼90 °C and stirred at 300 rpm in a water bath until reaching a transparent solution. Then, the agarose solution was cooled down to ∼40 °C. To prevent the degradation of heat-sensitive luciferase, the 40 °C agarose solution was pipetted to a ∼26 °C solution containing MgSO_4_, EDTA, d-luciferin, and luciferase (prepared in Tris–HCl buffer at pH 8); resulting in a final biosensing cocktail temperature of ∼28 °C after mixing. Unless otherwise noted, after optimization of the reaction parameters, the final pregel cocktail contained: 2 g L^–1^ agarose, 0.01 M MgSO_4_, 0.001 M EDTA, 0.01 M d-luciferin, and 2.98 × 10^10^ LU mL^–1^ luciferase,. The ATP sample for method development and testing was prepared by pipetting 20 μL of 10 mM ATP solution onto a 4 mm diameter circle drawn on wax paper and then dried. Subsequently, a 10 μL aliquot of the spreadable biosensing pregel was pipetted for ATP detection. For specimen analysis, a 20–30 μL aliquot of the cocktail was pipetted onto the surface.

### Pregel Preparation for the Detection of Ascorbic Acid

To prepare the pregel cocktail with TM reagent for AA detection, the agarose-water mixture was first heated to ∼90 °C in a water bath until a transparent solution formed. Then, the agarose solution was cooled to ∼40 °C (Figure 1A). TM reagent solution, prepared using 0.1 M phosphate buffer (pH 7), was added to agarose gel at the concentration and pH determined during optimization. The final pregel cocktail contained 2 g L^–1^ agarose and 1 mM TM reagent at pH 7. The AA sample was prepared by pipetting 100 μL of 1 mM AA onto the surface of a 10 mm diameter circle drawn on wax paper, then drying at room temperature for method development and reaction parameter optimization. Subsequently, 200 μL of TM reagent pregel mixture were dropped onto it to detect AA. For specimen analysis, a 3–5 mL aliquot of the cocktail was pipetted onto the surface.

### Plant Tissue Sample Preparation

Plants were purchased from local markets in Hsinchu, replanted on potting soil, and grown for two to 4 weeks to promote development of new leaves (width, 1–2 cm; length, 1–5 cm). The plants were grown under a full-spectrum LED plant growth lamp (25 W, 110 V; luminous flux, 5000 lm; luminous efficiency, 3400 lm W^–1^; Sanan Optoelectronic, Xiamen, China), providing an illuminance of 10,000 ± 500 lx to the plant canopy (∼20 cm from the light source). The lower epidermal leaf tissue was collected following a previously published mechanical separation protocol,^52^ with some modifications (Figure 1B). In summary, two paper tape strips (NKS masking tape; thickness, 0.130–0.160 mm; temperature resistance, −2 to 150 °C; Naikos (Xiamen) Industrial Company, Fujian, China) labeled L and U were used to separate the lower and upper epidermises (Figure 1B­(i)). The abaxial (lower) epidermis was intended to adhere to the L strip, and the adaxial (upper) leaf tissue to the U strip.^52,53^ The edges of both tape strips were trimmed to match the size of the leaf. For example, the upper side of the leaf was fixed onto the adhesive U tape strip. Once attached to the adhesive U strip, the leaf was sandwiched by placing the L tape strip onto it. A vial was pressed and rolled over the tape-leaf sandwich repeatedly to ensure the leaf was sandwiched between the two tape strips. This step was conducted 5–15× (e.g., cilantro leaf) and 10–20× (e.g., parsley and spearmint leaves) depending on the leaf thickness. The L tape strip was then carefully removed, separating the abaxial (lower) epidermal tissue from the remaining leaf tissues (Figure 1B­(i)). The abaxial epidermal tissue on the L tape strip was fixed onto a glass slide for subsequent mapping experiments. Additionally, leaf tissue was visualized using a fluorescence microscope (Figure S1).

### Imaging System

Images were captured using an Olympus digital camera (EM1; Olympus, Tokyo, Japan) positioned vertically above an imaging chamber (Figures 1C and S2). The imaging chamber (length, 32 cm; width, 24 cm; height, 32 cm), constructed from black acrylic, was used during image acquisition to provide controlled illumination. A sample holder and an LED light strip (Golden Electronics, Hsinchu, Taiwan) were installed inside the chamber. The bioluminescence measurement was done inside the imaging chamber without any light source in a dark background. The camera’s optical axis was aligned perpendicular to the upper surface of the sample holder. Images were saved as JPEG files on a computer using the digital camera software (Olympus Capture, version 3.1). The distance from the sample surface to the outermost camera lens was ∼18 cm. For colorimetric detection, the exposure time was set to 20 s, with a relative aperture (*F*) of 5.6 and an ISO value of 4000, while the LED light intensity was maintained at ∼50 ± 10 lx. For bioluminescence detection, the camera was set to manual mode (M) with an exposure time of 6 s, a relative aperture (*F*) of 5.6, an ISO value of 4000, and a white balance of 5200.

### Data Analysis

Depending on whether the upper or lower leaf surface was initially exposed on the tape, the corresponding images of the peeled tissue were geometrically flipped using CorelDraw (version X6; Corel, Ottawa, ON, Canada) to ensure a consistent orientation among images derived from the same leaves. The images were cropped using ImageJ software (version 1.54g; National Institutes of Health, Bethesda, MD, USA) and processed with custom-written Python scripts (Python Software Foundation, Wilmington, DE, USA) specific for glucose, ATP, and AA detection. The Python scripts extract the average gray value, green value, and blue value from a selected region of interest in the image, with 2500 pixels designated for glucose and AA detection and 400 pixels for ATP detection. The extracted values range from 0 to 255. The gray values were used for glucose analysis, while the blue and green values from RGB were used for ATP and AA analysis, respectively. The data sets were then plotted using Origin (version 2018b; OriginLab, Northampton, MA, USA). For method optimization and characterization experiments (including linearity, selectivity, and stability), the gray value difference for glucose (i.e., difference between wax paper without glucose [G_0_] and with glucose [G]) and the green and blue values for ATP and AA, respectively, were averaged across replicates. Error bars in the graphs represent the standard deviations calculated from three replicates.

## Results and Discussion

### Pregel with Trinder Reaction

The detection and mapping of glucose was based on Trinder reaction (glucose oxidase-peroxidase method; Figure S3A). Before mapping glucose on the surface of plant leaf specimens, the spreadable biosensing pregels were optimized for the following parameters: contact time, enzyme concentration, enzyme concentration ratio, and 4-AAP concentration (Figure S4). The highest signal was observed at a 5 min reaction time, with 5 U mL^–1^ GOx, a 1:1 ratio of GOx and HRP, and 50 mM 4-AAP. In an initial test, the analyte displacement from the sampled zones was verified (Figure S5). Glucose did not disperse from the small circles (diameter ∼ 5 mm) to the larger area in certain regions. After 5 min, the pink color appeared mainly in the small circles, indicating only minor analyte displacement despite the spreadable nature of the pregel mixture. The small circles with glucose on the mockup specimen developed a pink coloration, indicating the potential for mapping the spatial distribution of a target analyte on the specimen surface using the developed approach.

The analytical performance of the method was characterized prior to real-sample glucose detection. Gray-value responses of the biosensing gel were measured on wax paper coated with glucose at concentrations in the range of 1.27 × 10^–10^ to 6.37 × 10^–9^ mol mm^–2^. The calibration parameters—including the calibration equation, limit of detection (LOD), and coefficient of determination (*R* ^2^)—are summarized in Table S1. The selectivity of the method was evaluated by comparing the gray value of glucose (1 mM) against potential interfering substances such as sucrose, fructose, urea, and AA in leaves (Figure S6A). As expected, the method is selective toward glucose. Further, the stability of the spreadable biosensing pregel was examined by measuring the gray value of glucose coated on wax paper over 7 days. The pregel was prepared at room temperature and subsequently stored at ∼4 °C. Measurements were performed at 25–30 °C. The biosensing gel performance remained stable, with relative standard deviation (RSD) of 4.94% over 7 days of storage.

The Trinder reaction is a colorimetric method for glucose determination. The application is limited when plants endogenously produce hydrogen peroxide in response to abiotic and biotic stress.^54–56^ To evaluate this matrix effect, we conducted an experiment wherein the abaxial leaf epidermis was cut in half, with pregel containing GOx applied to one-half and pregel without GOx applied to the other half. Comparison of biosensing responses with and without GOx showed that glucose-related signals could be distinctly differentiated in cilantro leaves (Figure 2). Parsley and spearmint leaves exhibited indistinguishable hydrogen peroxide and glucose responses, indicating limited pregel biosensing selectivity due to matrix effects in the Trinder reaction (Figure S7). In future, another colorimetric reaction can be considered to mitigate this effect. Moreover, sucrose is generally the predominant soluble sugar in plants—serving as the main transport carbohydrate rather than glucose—while starch is an overflow product of carbon allocation in source leaves.^57,58^ Nevertheless, glucose remains a relevant metabolic intermediate,^16–18^ supporting the applicability of the pregel biosensing system in selected plant matrices.

**Figure 2:**
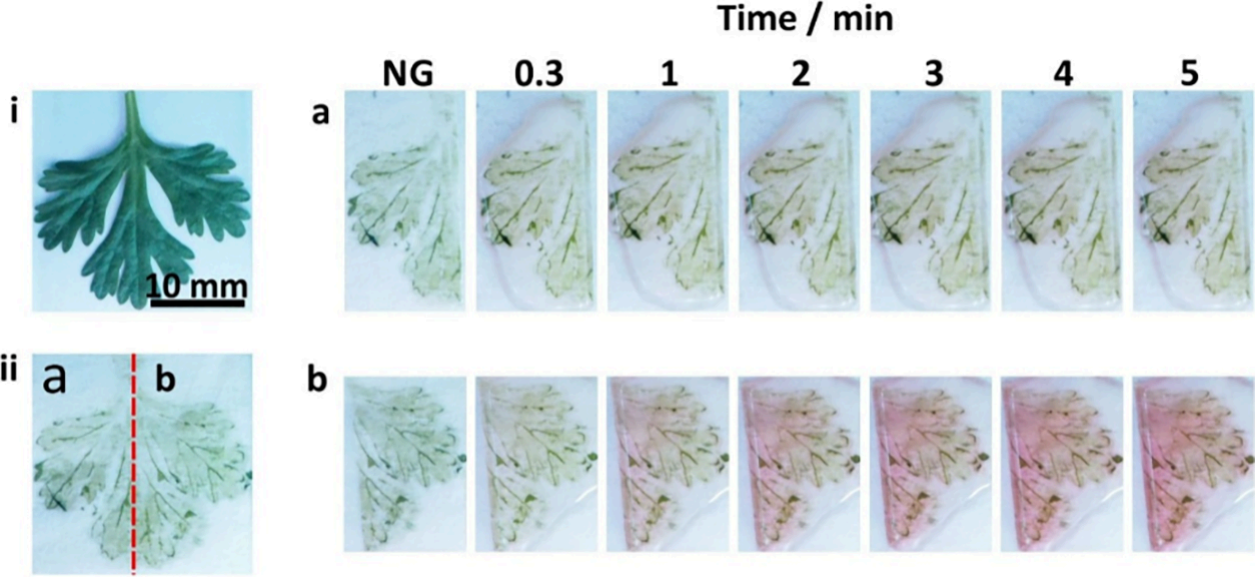
Colorimetric detection of hydrogen peroxide and glucose in cilantro leaf using HRP and GOx enzymes. (i) Raw cilantro leaf specimen; (ii) abaxial leaf epidermis cut into (a) and (b) before addition of biosensing pregel; images of a peeled leaf were captured after 5 min of contact time for both (a) abaxial leaf epidermis with biosensing pregel without GOx and (b) abaxial leaf epidermis with biosensing pregel. NG: no pregel added.

The current method can accommodate various specimen sizes and shapes. Subsequent advances have largely focused on immobilizing GOx within polymeric and hydrogel matrices to enhance analytical sensitivity and operational stability. Despite these technical improvements, most existing platforms are optimized for bulk or averaged glucose measurements and are inherently incapable of resolving spatial heterogeneity at biological interfaces.^59–61^ In contrast, the spreadable biosensing pregel method presented here addresses a critical methodological gap by enabling visualization of target metabolites directly on plant leaf surfaces.

### Mapping ATP Distributions

The buffer type was found to influence enzymatic performance and ATP stability, consistent with prior reports.^62,63^ Our results revealed distinct response differences between phosphate and Tris–HCl systems under pregel-confined conditions (Figure S8A). The use of Tris–HCl buffer (pH 8) led to an increased signal stability and high green pixel values, yielding more consistent and intensified response. The phosphate buffer produced less stable ATP-dependent signals, likely due to metal ion interactions and the buffer type impacting the activity of metal-dependent enzymes.^62^ In a previous study on solution-phase reactions, Nichols and co-workers recommend optimizing and standardizing buffer selection when characterizing new enzymes, particularly metalloenzymes, as the choice of buffer can substantially impact kinetic results.^64^ This emphasizes buffer chemistry as a critical parameter for hydrogel-based biosensing platforms, particularly during surface-mapping and long-duration measurements. For mapping ATP using the luciferase spreadable biosensing pregel, we used the stable Tris–HCl buffer system.

The detection and mapping of ATP was based on the luciferin-luciferase reaction (Figure S3B). The reaction was optimized with luciferin substrate, magnesium sulfate, and luciferase at concentrations of 1 × 10^–6^ to 1 × 10^–2^ M, 1 × 10^–5^ to 1 × 10^–1^ M, and 2.98 × 10^7^ to 2.98 × 10^10^ LU mL^–1^, respectively (Figure S8B–D). The optimum concentrations were 0.01 M for luciferin and magnesium sulfate, and 2.98 × 10^10^ LU mL^–1^ for luciferase, with stable bioluminescence up to 60 s (Figure S8B–E). The concentration of EDTA and pH were based on a previous study.^65^ We assessed the stability of the method by measuring the green value intensity of ATP over 7 days (Figure S9A). The enzyme pregel was prepared at room temperature and then refrigerated (∼4 °C). Measurements were taken at room temperature (25–30 °C) three times on each day, detecting ATP on wax paper (7.96 × 10^–9^ mol mm^–2^). The ATP measurements were reproducible, with an RSD of 8.25% (Figure S9A), demonstrating stable biosensing performance. To evaluate the quantitative capabilities of the method, we prepared a calibration plot for different ATP concentrations (1.59 × 10^–10^ to 1.59 × 10^–8^ mol mm^–2^; 8 levels) on wax paper (Figure S9B). The calibration parameters, including the calibration equation, LOD, and *R* ^2^, are summarized in Table S1. Repeatability was evaluated by depositing 10.19 nmol mm^–2^ ATP on wax paper and on marked regions (∼5 mm diameter circle) of abaxial leaf epidermis prior to bioluminescence measurement using the spreadable biosensing pregel (Figure S10). The detection of ATP on wax paper showed an RSD of 2.24% (*n* = 10). The RSD values for each of the spiked peeled leaves (Figure S10B­(i–iii)) were 5.17, 11.53, and 17.86% (*n* = 6), respectively, while the overall RSD for all three spiked leaves was 19.41% (*n* = 18).

ATP is a primary carrier of chemical energy in living organisms and is crucial for cellular metabolism.^66^ The variations in ATP distribution in leaf tissues can be influenced by metabolism,^67^ development, signaling, and stress,^66,68^ which are collectively responsible for forming gradients relative to surrounding cells. Therefore, the next experiment involved visualization of ATP in peeled leaves obtained from various plant species (cilantro, spearmint, and parsley; Figures 3 and S11). The method is similar to the one used for glucose mapping. The reagents for the luciferin-luciferase reaction were mixed with agarose pregel (2 g L^–1^) and spread directly on the peeled lower leaf surface. The results from analyzing cilantro, spearmint, and parsley leaves showed average green pixel values 35.82 ± 40.07, 63.98 ± 63.93, and 49.52 ± 23.48, respectively, for 3 replicate measurements taken on different days (Figures 3 and S11). The calculations were based on a 50 × 50-pixel square region selected approximately at the center of each leaf. Validation parameters cannot be obtained in experiments using real specimens due to the absence of blank leaf tissue matrix: leaf cells already contain glucose, hydrogen peroxide, ATP, and other metabolites. Despite this restriction, in another control experiment, a spearmint leaf was peeled, and the abaxial part was divided into small circles. Each circle was spiked with ATP at different concentrations (1.02, 4.08, and 8.15 nmol mm^–2^). After drying, the circles were coated with a biosensing pregel containing luciferase. Bioluminescence was captured by a camera. The green pixel values of three unspiked abaxial leaf circles (5 mm in diameter) were visualized, showing the difference between the unspiked and spiked circles (Figure S12).

**Figure 3:**
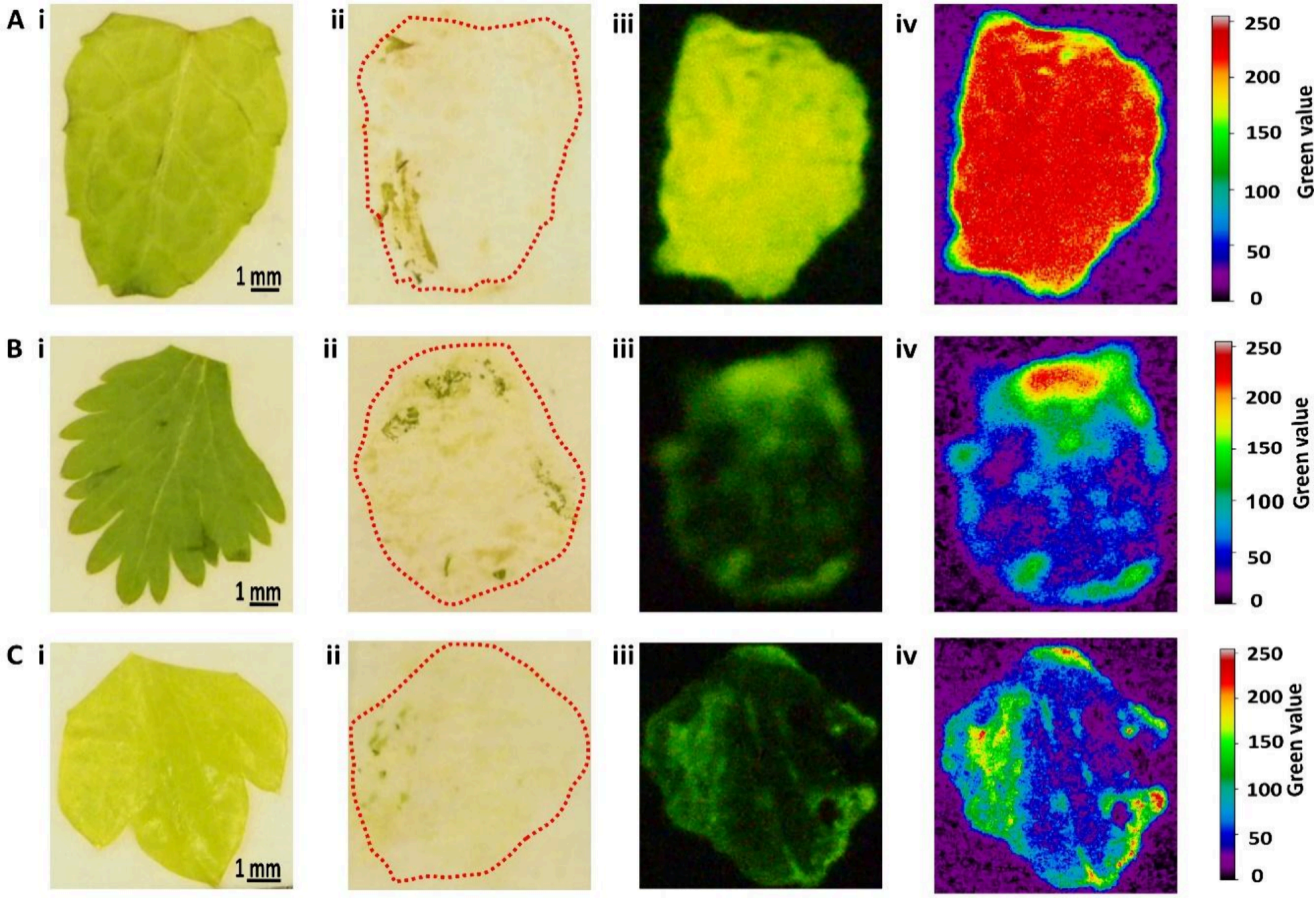
ATP distribution on the abaxial leaf epidermis detected and mapped using spreadable biosensing pregel: (A) spearmint leaf; (B) cilantro leaf; (C) parsley leaf. Columns (i)–(iv): (i) leaf attached on the paper tape strip, (ii) abaxial leaf epidermis without spreadable biosensing pregel; red dashed outline indicates the detection region, (iii) bioluminescence light generation after 25-s contact time with spreadable biosensing pregel, (iv) spatial distribution of ATP mapped as green value intensity.

For pregel optimization, characterization, and an additional application, please see the Supporting Information.

## Conclusions

We have demonstrated the feasibility of the spreadable biosensing pregel approach, in which the bioreagents are blended with a viscous pregel matrix. Such a biosensing blend can be directly applied onto surfaces of solid objects for rapid rudimentary visualization of analyte distributions. The gel matrix plays a role of dispersing medium, anticonvective medium, enables enzyme immobilization and stabilization through entrapment, ensures presence of sufficiently thick layer of the reaction mixture on top of the specimen, and minimizes water evaporation. The method was successfully implemented in imaging of glucose, ATP, and AA, in biological specimens. The approach can readily be combined with separated plant tissues (leaves) enabling rapid sensing of biochemicals. Since the pregel matrix is biocompatible, it can potentially accommodate a variety of enzymes and other bioreceptors, which can further enhance analyte coverage. Unlike conventional plant tissue imaging techniques that require tissue sectioning, bulk extraction, or specialized instrumentation, the spreadable biosensing pregel can be directly applied to intact surfaces with minimal sample preparation. The limitations of this approach are the limited spatial resolution due to the possible dispersion of surface analytes during pregel application, blurring of the concentration gradients due to diffusion and convection, reflection of light within the pregel layer, and the limited quantitative capabilities. Although a matrix effect was observed in glucose sensing due to the presence of endogenous hydrogen peroxide, the method can easily be adapted for sensing hydrogen peroxide – a stress marker in plants. As a further consideration, direct comparison of results obtained with the spreadable biosensing pregel and conventional techniques is not feasible because the method maps analytes on intact 2D surfaces, whereas standard methods extract analytes from bulk 3D tissues and provide quantitative data without preserving spatial information. While multiplexed detection of analytes would be appealing, currently it is not possible due to the expected interference between the chromogenic species and/or light emitted in different assay reactions. The advantages of the approach—such as mapping capability, simplicity, stability, user-friendliness, and speed—outweigh those limitations making the method suitable for various potential (semi)­quantitative applications. This proof-of-concept study demonstrates the feasibility of a spreadable biosensing pregel for mapping chemical distributions on surfaces, with potential applicability to emerging areas such as precision agriculture, where rapid characterization of spatial heterogeneity may be beneficial.

## Supplementary Material



## Notes

Additional experimental details, including biosensing reactions, visualization of peeled leaf using fluorescence microscope, and characterization of biosensing pregel; additional results and discussion, incuding optimization and characterization of soft biosensing pregel and mapping ascorbic acid distributions in sliced fruits; additional table (S1): calibration parameters for glucose, ATP, and AA detection; additional figures (S1–S23): Visualization of cilantro leaf tissue using a fluorescence microscope, photograph of the imaging setup, enzymatic and chemical reaction, optimization of glucose biosensing, evaluation of analyte displacement using wax paper, characterization of biosensing pregel performance for glucose detection, colorimetric detection of hydrogen peroxide and glucose in mint and parsley leaves, optimization of ATP biosensing, characterization of biosensing pregel performance for ATP detection, repeatability of ATP detection, replicates of ATP detection and mapping, bioluminescence of spiked and unspiked abaxial leaf epidermis of spearmint leaves, effect of agarose concentration on biosensing pregel performance; influence of agarose concentration on dispersion of reaction product and enzyme activity, evaluation of pregel uniformity, evidence for homogeneous mixing of agarose and other components, rheological properties of the 2 g L^–1^ agarose, stability of the biosensing cocktail at freeze-thaw condition, optimization of AA sensing, characterization of sensing pregel performance for AA detection, AA detection and mapping on sliced citrus fruit tissue, comparison of lemon slice specimens without pregel (NG) and with pregel not containing TM reagent, and comparison of AA distribution between fresh lemon fruit slices and slices dried at 40 °C and 60 °C; additional references; and computer codes (PDF)
