# Supplementary material for: Spreadable Biosensing Pregel for Analyte Visualization in Peeled Plant Tissues
Source: Anal Chem. 2026 Jul 6;98(28):20716–24. doi: 10.1021/acs.analchem.6c01240 (PMC13393083; doi:10.1021/acs.analchem.6c01240)
Supplement: Supplementary file 1 [file ac6c01240_si_001.pdf]

SUPPORTING INFORMATION

Spreadable Biosensing Pregel for Analyte Visualization in Peeled Plant Tissues

Hayelom Dargo Beyene, Decibel P. Elpa, Pawel L. Urban*

Department of Chemistry, National Tsing Hua University

101, Section 2, Kuang-Fu Rd., Hsinchu, 300044, Taiwan

* Corresponding author:

P.L. Urban (urban@mx.nthu.edu.tw)

Table of Contents

Additional experimental details……………………………………………………………S-2

Additional results and discussion…………………………………………………………….S-3

Additional table…………………………………………………………………………S-6

Additional figures……………………………………………………………………….S-7

Additional references…………………………………………………………….………S-30

Computer codes…………………………………………………………….………….S-32

ADDITIONAL EXPERIMENTAL DETAILS

Biosensing reactions

For glucose detection, the imaging biosensor utilized an enzymatic reaction, specifically the Trinder reaction.[1] The sensing mechanism relied on the oxidation of glucose by GOx, generating gluconic acid and hydrogen peroxide. In the presence of HRP, the hydrogen peroxide reduces the 4-AAP and phenol to generate a pink color *N*-antipyridine-*p*-benzoquinoneimine product (**Fig. S3A**).

The ATP detection was based on luciferin-luciferase reaction (**Fig. S3B**). The reaction proceeds in two steps: first, luciferase's substrate, luciferin, reacts with ATP-Mg^{2+} to form inorganic pyrophosphate and an intermediate called luciferyl-adenylate; then, luciferyl-adenylate is oxidized and decarboxylated to oxyluciferin, which emits yellow-green light (550–570 nm), producing CO_2, AMP, and photons.[2]

The ascorbic acid (AA) detection was based on the redox reaction between AA and 2,6-dichlorophenolindophenol (DCPIP; **Fig. S3C**). AA reduces DCPIP from its oxidized blue form to the colorless $DCPIPH_2$, while AA is oxidized to dehydroascorbic acid.[3]

Visualization of peeled leaf using fluorescence microscope

A 0.05% TBO staining solution was prepared by dissolving 5 mg of TBO in 100 mL of 0.1 M phosphate buffer (pH 6.5).[4] The solution was filtered through filter paper and stored at 4 °C in an amber bottle to prevent light-induced degradation. For staining, the peeled lower part leaf was placed on a glass slide, and 1-2 drops of the TBO solution were added. The sample was incubated for 5-15 min, depending on tissue thickness, and the glass slide was then gently rinsed with distilled water and followed by phosphate buffer. Finally, the sample was mounted under a coverslip with a suitable medium and examined using a fluorescence microscope.

Characterization of biosensing pregel

The rheological properties of the pregel were measured at room temperature (~ 25 °C) using a rheometer (MCR302e; Anton Paar, Graz, Austria) with cone-plate geometry (diameter, 50 mm; cone angle, 1°). Viscosity was measured as a function of shear rate while storage modulus and loss modulus were measured as a function of angular frequency.

ADDITIONAL RESULTS AND DISCUSSION

Optimization and characterization of soft biosensing pregel

The effect of agarose concentration was evaluated. Pore size has been reported to decrease with increasing agarose concentration (*cf*. refs.[5,6]). Here, the results showed that agarose concentrations from 1-5 g L^{-1} did not affect glucose detection or enzyme activity (**Fig. S13**). A concentration of 2 g L^{-1} was selected because it has the appropriate texture which enabled its easy application onto the specimen surface. We also evaluated the influence of agarose concentration on analyte displacement. Images of the pregel biosensing showed that most of the deposited glucose exhibited only minor analyte displacement and remained localized, as indicated by the pink coloration confined within the small circles after 5 min of pregel contact (**Fig. S14**). This suggests that, within the evaluated agarose concentration range (1-5 g L^{-1}) and at the applied glucose loading (2.55×10^{-9} mol mm^{-2}), agarose concentration has no observable effect on analyte displacement.

The biosensing pregel was then characterized in terms of its physicochemical properties and rheological behavior. The thickness of the pregel was estimated based on the deposited gel mass, specimen surface area, and gel density (0.966 mg mm^{-3}). For example, a 200 µL glucose pregel cocktail pipetted onto a 100 mm^2 surface area and a 30 µL ATP pregel cocktail pipetted onto a 25 mm^2 surface area were estimated to have ~ 2 mm and ~ 1 mm thickness for glucose and ATP measurements, respectively. To evaluate pregel thickness uniformity, 600 µL of the pregel spiked with 1 mM methyl red was applied onto the surface of a peeled leaf specimen. After a 5-min contact time, the sample was gently covered with a paper towel to visualize thickness uniformity across the pregel surface. A uniform red coloration was observed on the paper towel, without any visible uncolored or darker regions, indicating a homogeneous pregel thickness across the leaf surface (**Fig. S15**). The exact diffusion coefficients for the tested analytes were not determined. However, literature reports indicate that low-molecular-weight species in agarose hydrogels generally exhibit diffusion coefficients in the range of ~ 10^{-6} cm^2 s^{-1} under comparable conditions.[7,8] Since glucose, ATP, and AA are all relatively small molecules, substantial differences in their diffusion behavior within the pregel matrix are not anticipated. In this work, the "one-pot" preparation results in a homogeneous mixture, as verified using methyl red dye (**Fig. S16**). The enzyme is directly incorporated and uniformly dispersed throughout the pregel cocktail. No enzyme is lost during preparation, and additional steps to remove unbound enzymes on solid supports or high-viscosity gels are not required. Thus, the method can be considered "wasteless".

Rheological measurements showed that the viscosity of the biosensing pregel decreased from 9140.0 to 9.5 mPa·s with increasing shear rate from 0.1 to 100 s^{-1}, indicating shear-thinning behavior (**Fig. S17A**). Measurements of the storage and loss moduli (**Fig. S17B**) further showed that the pregel exhibited solid-like behavior over an angular frequency range of 0.1-237 rad s^{-1}, and transitioned to liquid-like behavior at higher angular frequencies (422-628 rad s^{-1}). The spreadable biosensing pregel remained in a sol-like state during ATP and AA detection at room temperature without forming a percolated hydrogel network, which is considered a criterion for gel formation.[9] However, during glucose detection at room temperature, a soft gel formed after 5 min.

All pregel biosensing was performed at room temperature, and the pregel cocktail was not exposed to freezing conditions at any stage. To evaluate performance under temperature variations, the biosensing pregel was subjected to three freeze–thaw cycles prior to glucose detection (**Fig. S18**). The average gray value was 88.65 ± 2.80, with an RSD of 9.11%, demonstrating stable and robust performance under these conditions.

Mapping ascorbic acid distributions in sliced fruits

The pregel is mixed with TM reagent for mapping of AA in the cross section of sliced fruits. Detection and spatial mapping of AA were achieved through its reaction with the TM reagent (**Fig. S3C**). Optimization of reaction conditions within the reagent cocktail revealed that a pH of 7, a TM reagent concentration of 1 mM, and a contact time of 1 min produced the highest decolorization response (**Fig. S19A–C**). Under these conditions, the spreadable pregel–TM reagent system showed high selectivity for AA, with negligible interference from flavanones (hesperidin, naringenin), phenolic acids (caffeic acid, sinapic acid), and reducing sugars (glucose, fructose) commonly found in citrus fruits (**Fig. S20A**). The sensing pregel maintained a consistent blue value response over 7 days, with RSD of 9.70% when stored at ~ 4 °C and measured at room temperature (**Fig. S20B**). The system demonstrated sensitivity in the low micromolar range and facilitated rapid diffusion of the TM reagent. A linear calibration response was obtained for AA-coated wax paper in the range of 1.27×10^{-10} to 6.37×10^{-9} mol mm^{-2} (**Fig. S20C**), with calibration parameters summarized in **Table S1**.

Mapping the distribution of AA is crucial for understanding nutrient localization and ripening behavior in fruits. Previous methods—such as electrochemical detection with multiwall carbon nanotubes/polyaniline-modified gold electrodes,[10] and etched fiber-based plasmon sensor,[11] and smartphone-read microneedle patches[12]—offer sensitivity but are limited by spatial resolution, or the need for specialized equipment. Paixão and co-workers demonstrated that a positionable platinum disk microelectrode effectively measures spatial distribution of AA concentration in cut orange fruit.[13] They explored the use of the microelectrode sensor to measure AA concentration *in situ* by inserting a platinum microelectrode at a depth of 5 mm into an orange tissue. This underscores the need for a simple method for producing spatially resolved AA maps on fruit tissues.

We have adapted the spreadable pregel with TM reagent for spatial mapping of AA on the surface of dried citrus fruit tissue sections (**Fig. S21**). A control experiment for pregel sensing of AA in fruit slices confirmed that, in the absence of the TM reagent, no reaction or decolorization occurred (**Fig. S22**). The TM reagent mixture was spread on dry fruit slices for localized detection of endogenous AA, producing a quantifiable colorimetric signal and enabling *in situ* imaging without sample extraction (**Fig. S21**). Fruit slices were pre-dried prior to analysis to reduce moisture-related matrix effects and improve detection. It should be noted that drying may cause degradation of AA. Thus, the detected signal reflects the remaining AA after drying rather than the original concentration in fresh samples. AA mapping showed heterogeneous distribution across the dried fruit cut perpendicular to the longitudinal axis. This may be attributed to drying-induced nonuniform surfaces that caused uneven contact with the reagent mixture. Furthermore, high AA levels were observed in the outer mesocarp, consistent with a previous report for orange fruit.[13] Detection with the TM reagent sensing pregel enabled the generation of AA maps for dried lemon, kiwi, and orange

slices. Spatial differences were noted among the fruits, with kiwi showing the highest TM decolorization by AA, followed by lemon and orange. Fresh citrus fruit slices and citrus fruit slices dried at 40 °C and 60 °C were also evaluated (**Fig. S23**). However, direct comparison among the three conditions was limited due to matrix effects, including high moisture content, which affected decolorization during reaction with TM reagent. Overall, the sensor provides a simple method for AA mapping and offers insights into nutrient distribution patterns on fruit surfaces.

ADDITIONAL TABLE

Table S1. Calibration parameters for glucose, ATP, and AA detection using the spreadable (biosensing) pregel. LOD was calculated using the formula: *LOD* = (3 × *standard error of y-intercept*)/*slope*.

Analyte	Calibration equation	R^2	LOD / mol mm^{-2}
Glucose	$G_0 - G$ = (4.47×10^{9} ± 0.34×10^{9})C + (46.46 ± 1.30)	0.978	8.73×10^{-10}
ATP	*Green value* = (1.45×10^{10} ± 9.78×10^{8})C + (12.21 ± 8.20)	0.973	1.70×10^{-9}
AA	*Blue value* = (1.34 ×10^{10}±1.42 ×10^{9})C + (58.23 ± 5.07)	0.947	1.14×10^{-9}

ADDITIONAL FIGURES

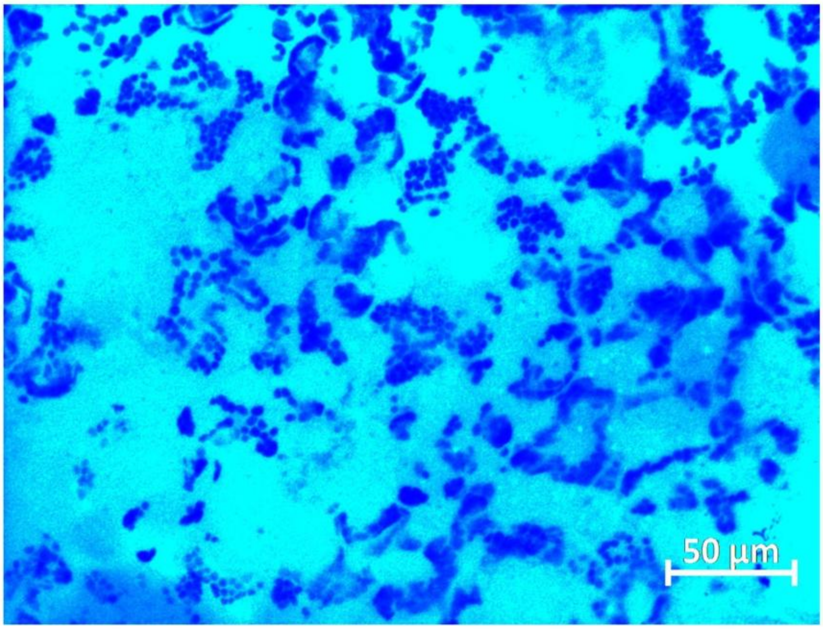

Figure S1. Visualization of cilantro leaf tissue using a fluorescence microscope (Axio Imager M2; Carl Zeiss Microscopy, Jena, Germany; objective, 20×; exposure time, 50 ms; light source, X-Cite mercury/metal halide lamp, model XI120-Q; excitation wavelength, 332-375 nm; emission wavelength, 435-485 nm). The abaxial epidermis of cilantro leaf was stained using 0.05% TBO solution.

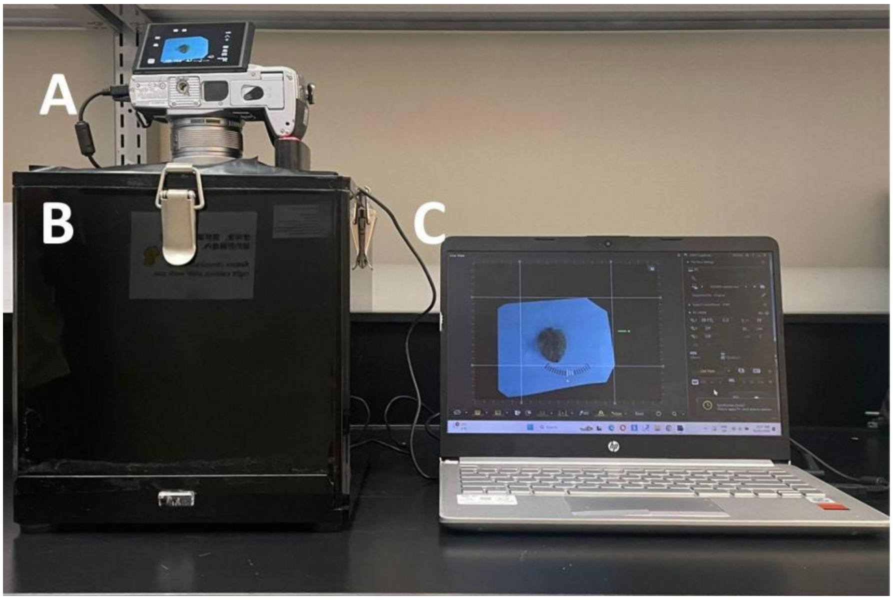

Figure S2. Photograph of the imaging setup: (A) camera; (B) imaging chamber; (C) computer.

A glucose + O_2 $\xrightarrow{GOx}$ gluconic acid + H_2O_2

H_2O_2 + 4-aminoantipyrine + phenol $\xrightarrow{HRP}$ quinoneimine (pink) + H_2O

B ATP + luciferin $\xrightarrow{luciferase,\ Mg^{2+}}$ luciferyl adenylate +PPi

luciferyl adenylate + O_2 $\longrightarrow$ oxyluciferin + AMP + light

C ascorbic acid + DCPI (blue) $\longrightarrow$ dehydroascorbic acid + $DCPIH_2$ (colorless)

Figure S3. Enzymatic and chemical reaction: (A) glucose-glucose oxidase reaction with HRP and 4-AAP for colorimetric detection of glucose on leaf surface; (B) luciferin-luciferase reaction for bioluminescence detection of ATP on leaf surface; (C) reduction of 2,6-dichlorophenolindophenol (DCPIP) by AA used as a test reaction for detection of AA on fruit tissue.

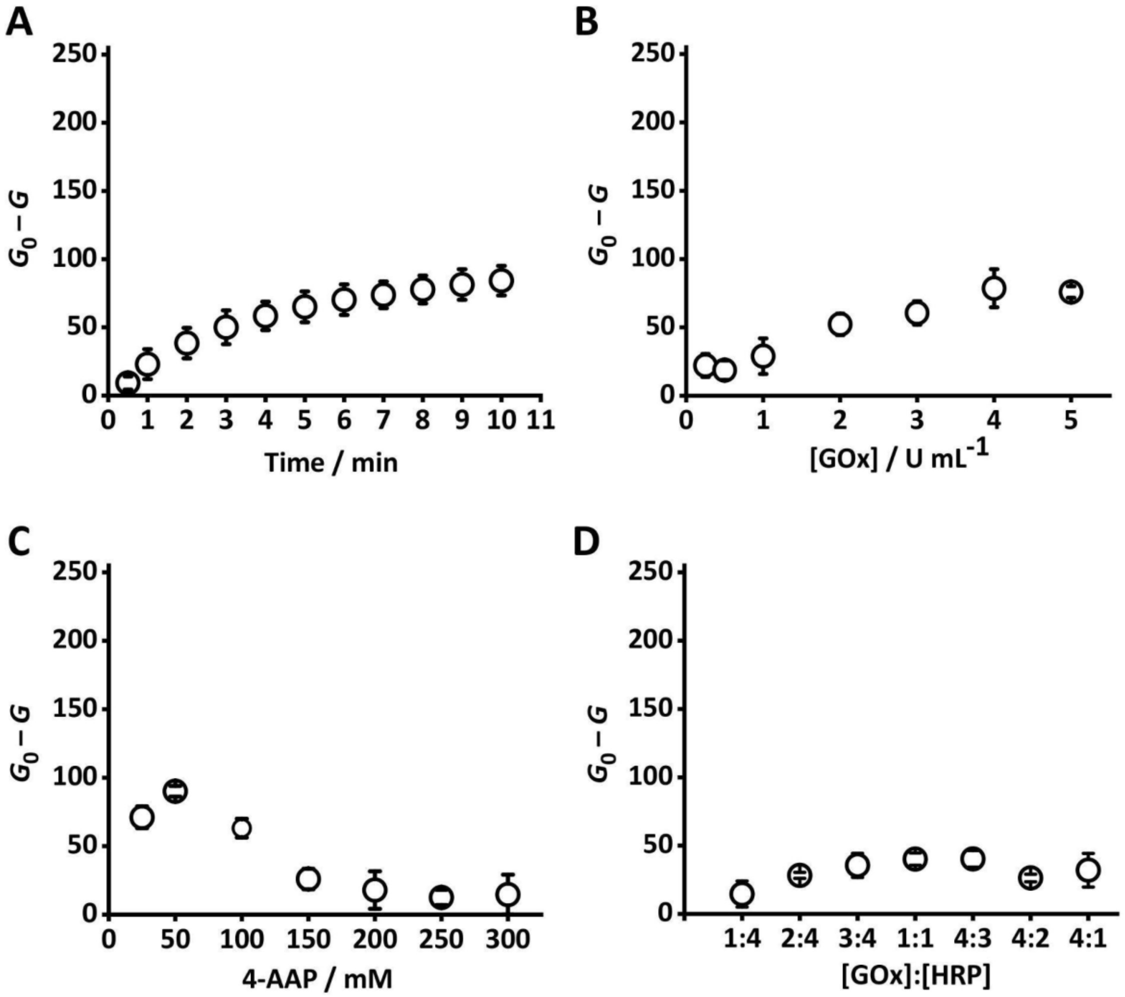

Figure S4. Optimization of glucose biosensing: (A) contact time optimization; (B) enzyme concentration optimization; (C) 4-AAP concentration optimization; (D) enzyme ratio optimization. The gray value difference (G_0-G) corresponds to the gray value from wax paper without glucose (G_0) and gray value from wax paper with glucose (G).

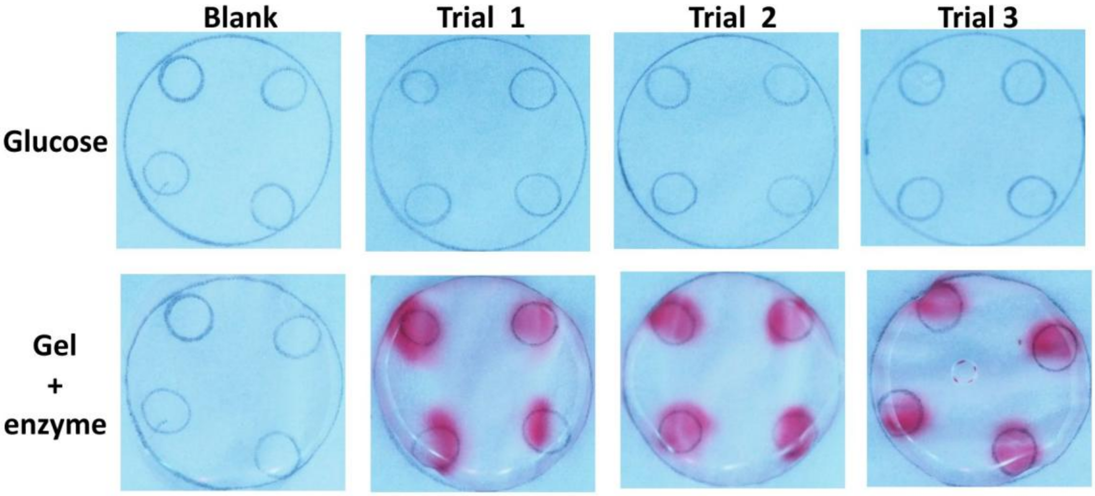

Figure S5. Evaluation of analyte displacement using wax paper with drawn 25-mm diameter circle, each with four drawn ~ 5-mm diameter inner circles: (i) 50 µL of 1 mM glucose loaded to each ~ 5-mm diameter circle; (ii) 1.5 mL of biosensing pregel pipetted over the 25-mm diameter circle. Photo of glucose detection after 5 min of biosensing pregel contact time.

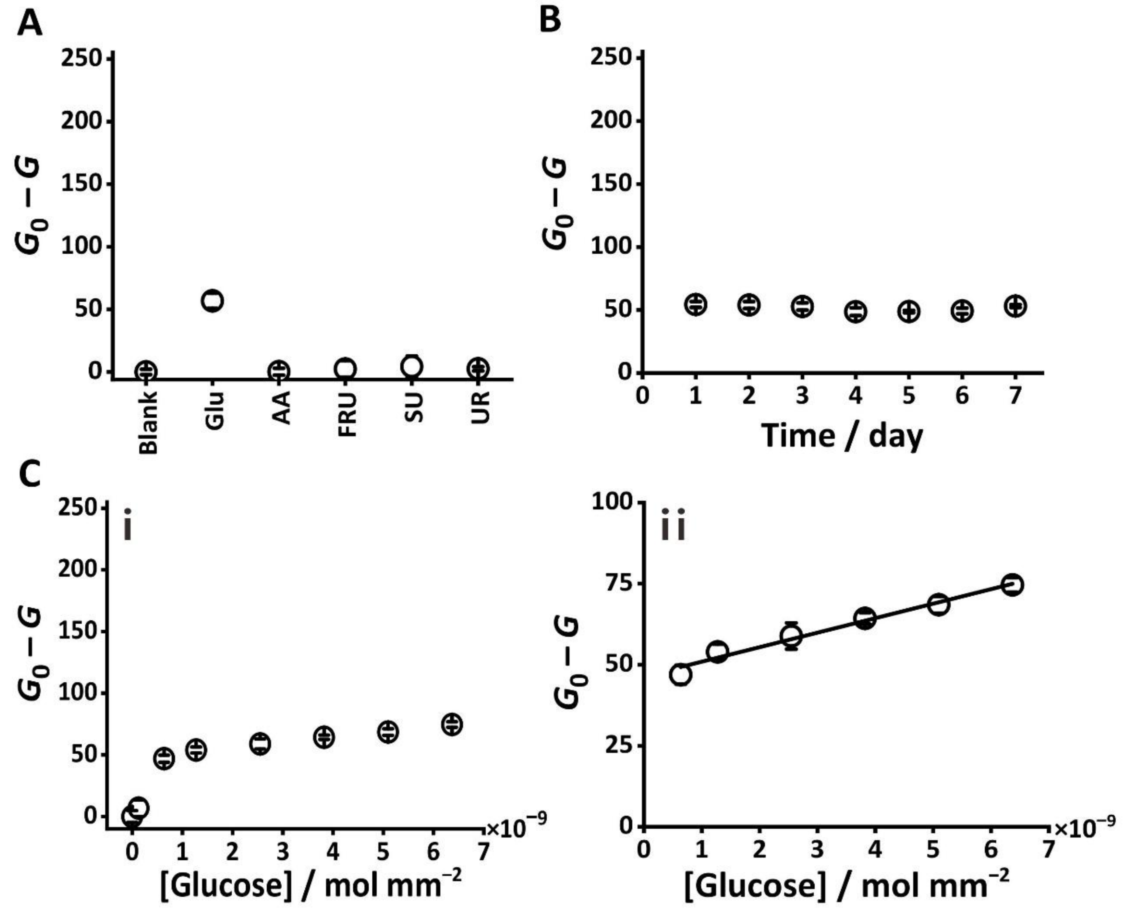

Figure S6. Characterization of biosensing pregel performance for glucose detection: (A) selectivity test for glucose (Glu) against potentially interfering analytes—ascorbic acid (AA), fructose (FRU), sucrose (SU), and urea (UR); (B) stability test—detection of 1.27×10^{-9} mol mm^{-2} glucose on wax paper for 7 days, n = 3 each day; (C) calibration curve: (i) glucose concentrations at 8 levels and (ii) linear response of 6 levels. The gray value difference (G_0-G) corresponds to the gray value from wax paper without glucose (G_0) and gray value from wax paper with glucose (G).

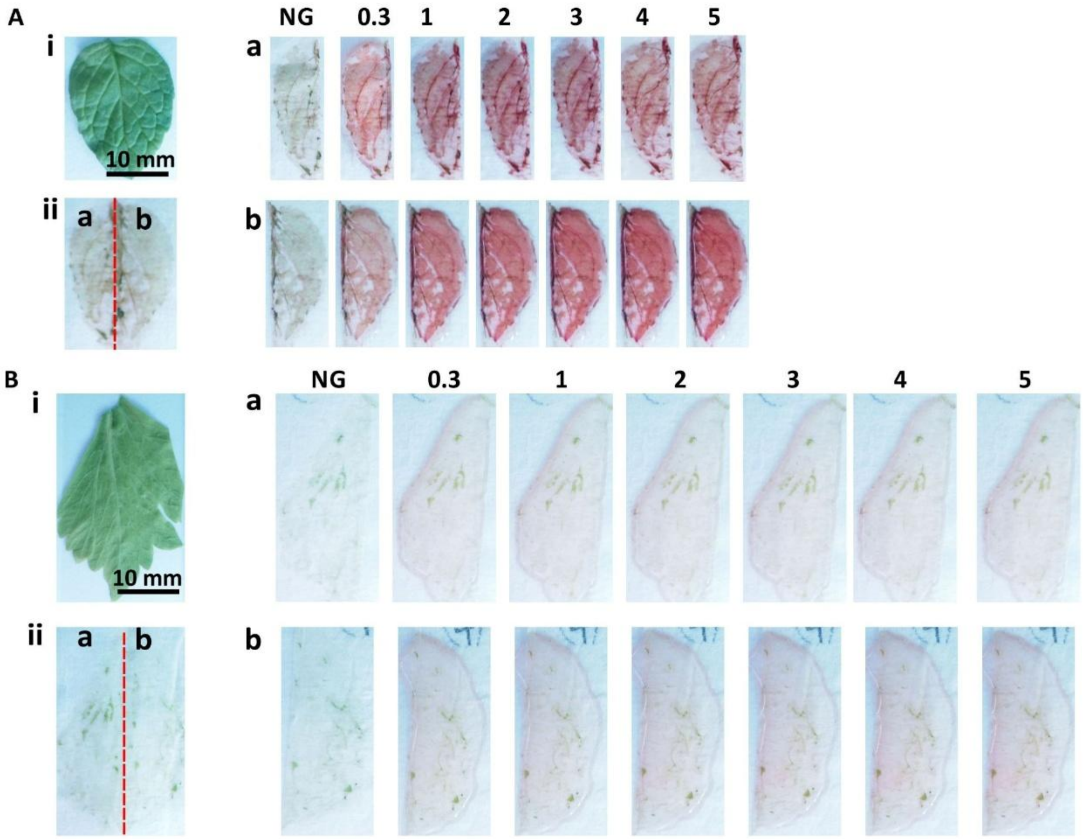

Figure S7. Colorimetric detection of hydrogen peroxide and glucose in (A) mint and (B) parsley leaves. (i) Raw leaf specimen; (ii) abaxial leaf epidermis cut into (a) and (b) before addition of biosensing pregel; images of a peeled leaf were captured for 5 min of contact time for both (a) abaxial leaf epidermis with biosensing pregel without GOx and (b) abaxial leaf epidermis with biosensing pregel. NG: no pregel added.

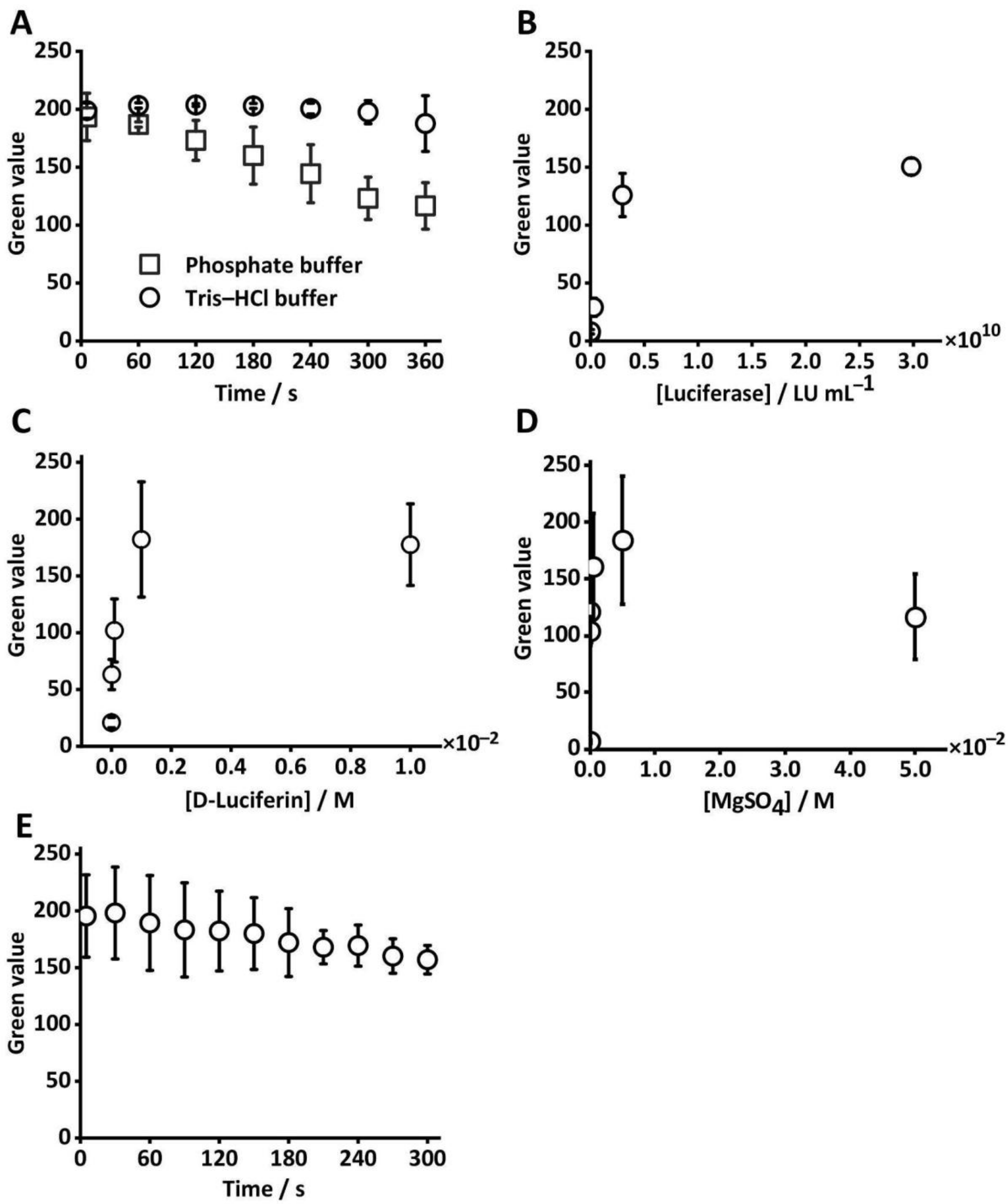

Figure S8. Optimization of ATP biosensing: (A) buffer optimization; (B) enzyme concentration optimization; (C) D-luciferin concentration optimization; (D) $MgSO_4$ concentration optimization; (E) contact time optimization.

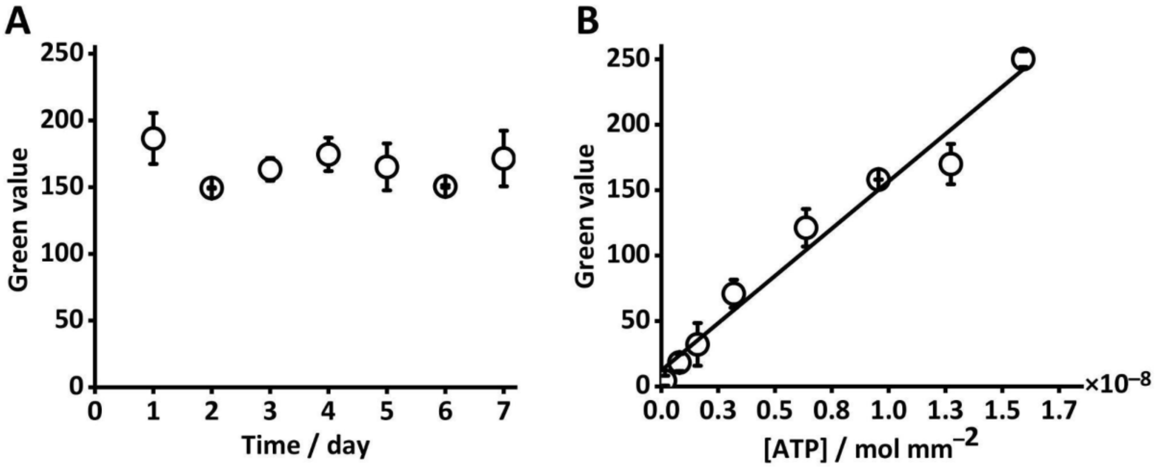

Figure S9. Characterization of biosensing pregel performance for ATP detection: (A) stability test—detection of 1.59×10^{-8} mol mm^{-2} ATP on wax paper for 7 days, n = 3 each day; (B) calibration curve.

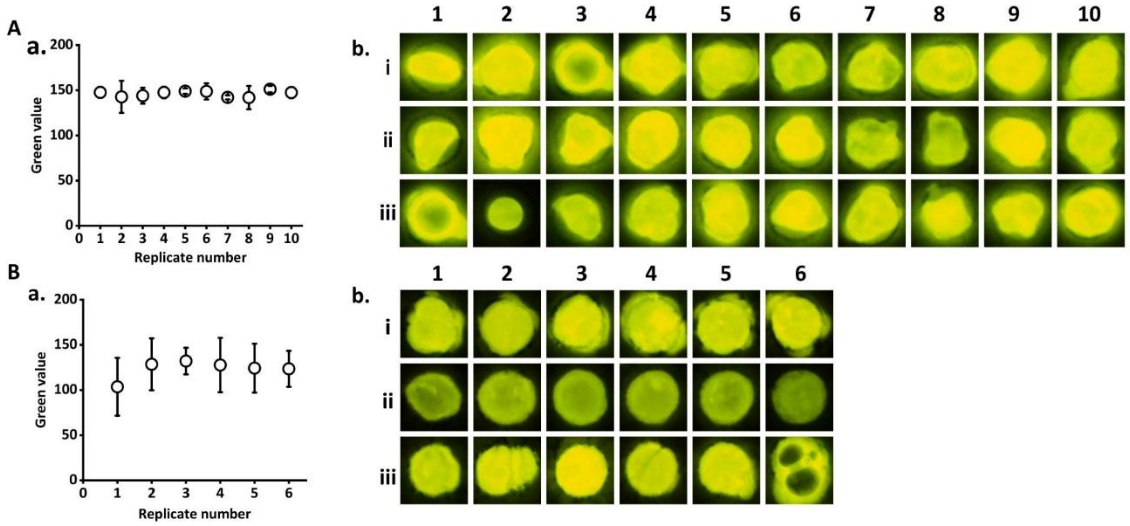

Figure S10. Repeatability of ATP detection. (A) Mock-up sample: (a) green values obtained for 10 replicates of mock-up samples prepared by depositing 10.19 nmol mm^{-2} ATP onto wax paper; (b) the corresponding bioluminescence images for 10 replicates. (B) Spiked spearmint leaf specimen: (a) green values obtained for 6 replicates of the abaxial leaf epidermis spiked with 10.19 nmol mm^{-2} ATP; (b) the corresponding bioluminescence images for 6 replicates. (i)–(iii) represent three technical replicates conducted for each sample and specimen.

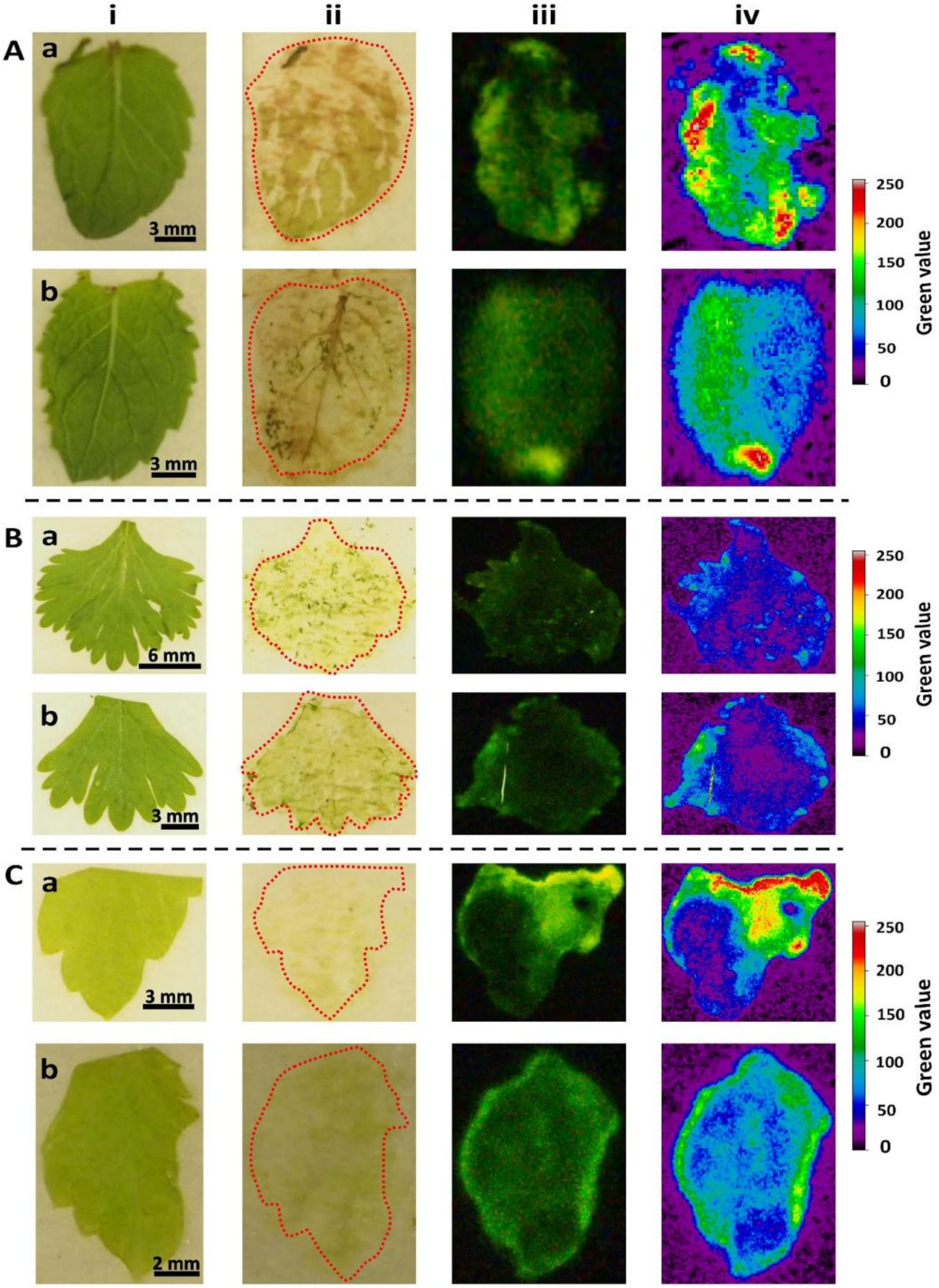

Figure S11. Replicates of ATP detection and mapping. Rows (a) and (b) represent replicates for ATP mapping for (A) spearmint leaves; (B) cilantro leaves; and (C) parsley leaves. Columns (i)–(iv): (i) photo of leaf attached on the paper tape strip, (ii) abaxial leaf epidermis without spreadable biosensing pregel; red dashed outline indicates the detection region, (iii) bioluminescence light generation after 25-s contact time with spreadable biosensing pregel, (iv) spatial distribution of ATP mapped as green value intensity.

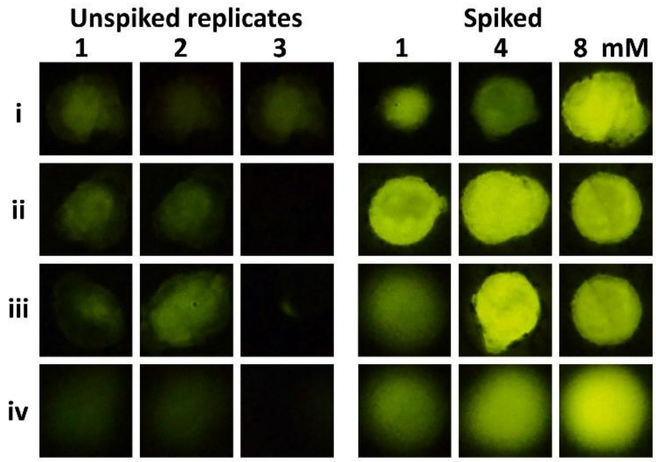

Figure S12. Bioluminescence of spiked and unspiked abaxial leaf epidermis of spearmint leaves. (i)-(iv) represent leaf specimens unspiked and spiked with 20 µL of ATP solution at different concentrations.

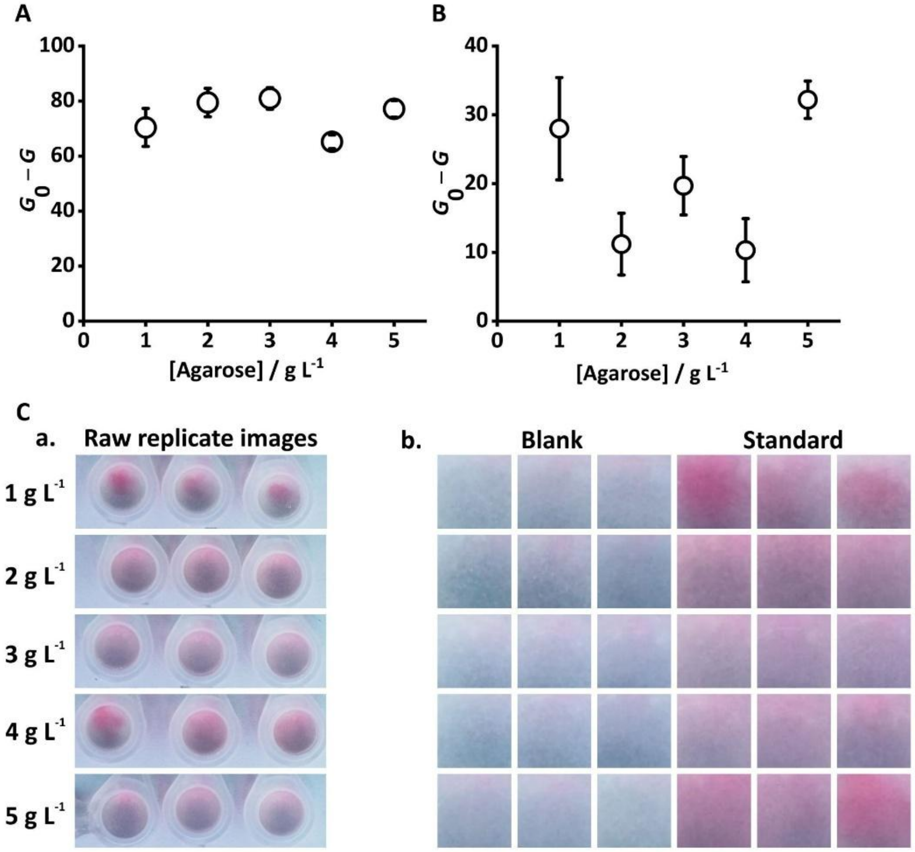

Figure S13. Effect of agarose concentration on biosensing pregel performance: (A) optimization of agarose concentration; (B) effect of agarose concentration on enzyme activity; (C) images of glucose detection after 5 min of contact time: (a) raw images showing glucose detection in microcentrifuge tube caps, where 200 µL of the biosensing pregel was pipetted, followed by addition of 50 µL of 1 mM glucose solution after 1 min; (b) cropped 2500-pixel image sections from the raw images, corresponding to the central region of each sample. The gray value difference (G_0-G) corresponds to the gray value from wax paper without glucose (G_0) and gray value from wax paper with glucose (G).

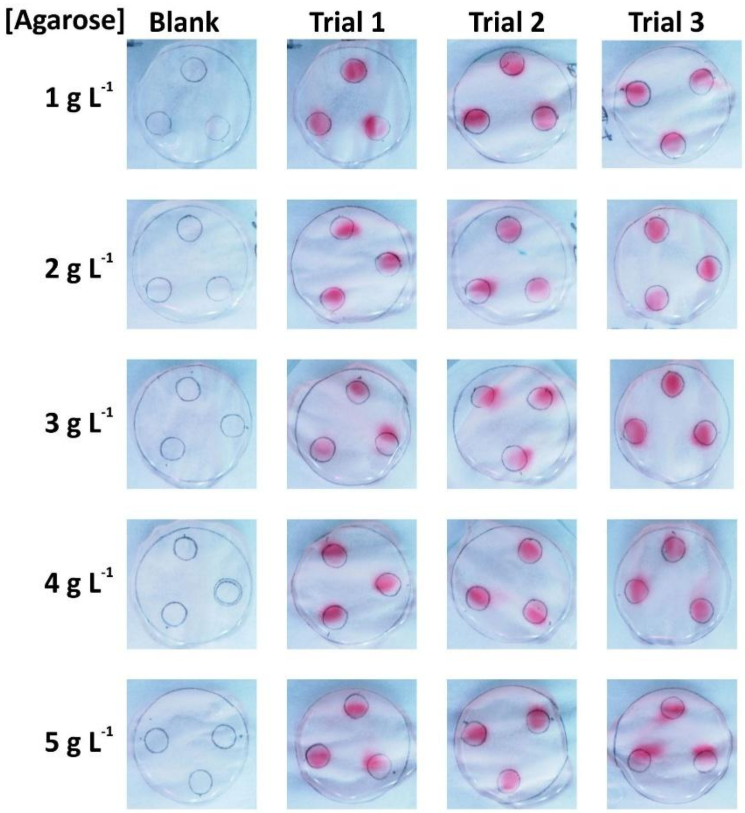

Figure S14. Influence of agarose concentration on dispersion of reaction product and enzyme activity for 50 μL of 1 mM glucose dispensed into each small circle. Blank represents the case without glucose.

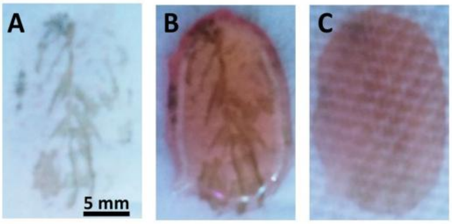

Figure S15. Evaluation of pregel uniformity: (A) abaxial spearmint leaf epidermis without spreadable biosensing pregel; (B) abaxial spearmint leaf epidermis coated with a pregel spiked with 1 mM methyl red after 5-min contact time; (C) visualization of pregel distribution by covering the gel layer with paper towel.

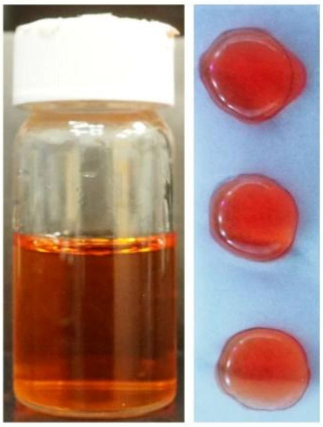

Figure S16. Evidence for homogeneous mixing of agarose (2 g L^{-1}) and other components during "one-pot" preparation of the biosensing cocktail using methyl red dye (1 mM). Preparation time: 5 min. Temperature: 40 °C.

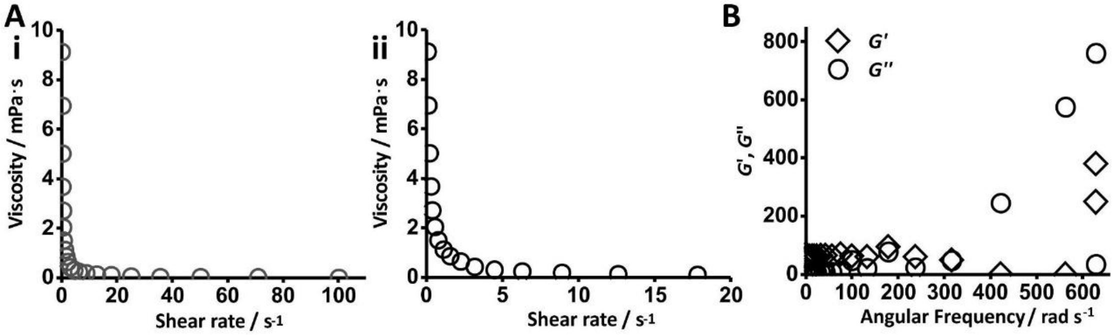

Figure S17. The rheological properties of the 2 g L^{-1} agarose: (A) viscosity; (B) storage (*G*′) modulus and loss (*G*′′) modulus.

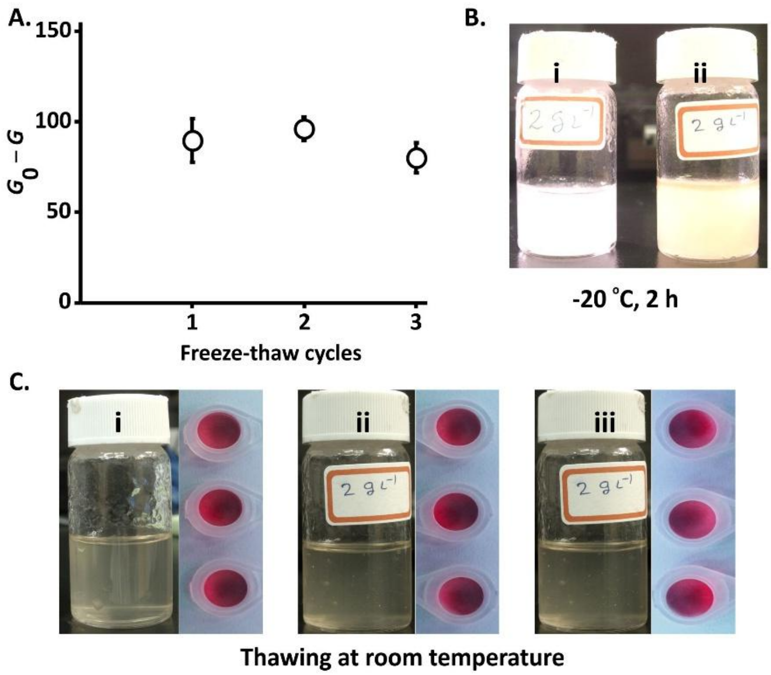

Figure S18. Stability of the biosensing cocktail at freeze-thaw condition: (A) gray values of the three freeze-thaw cycles; (B) pregel stored at -20 °C for 2 h: (i) pregel without enzymes and other reagents (4-AAP and phenol) and (ii) pregel with enzymes and other reagents; and (C) pregel subjected to three freeze-thaw cycles (i-iii) with 2 h intervals. The biosensing cocktail was kept at -20 °C for 2 h. Subsequently, it was thawed for 1 h at room temperature. For detection, 200 µL of the biosensing pregel were pipetted into microcentrifuge tube caps, followed by the addition of 50 µL of 1 mM glucose solution after 1 min. Images were captured after 5 min, and 2500-pixel image sections were cropped from the raw images, corresponding to the central region of each sample. This procedure was performed every 2 h three times to show the stability of pregel freeze-thawing. The gray value difference (G_0-G) corresponds to the gray value from wax paper without glucose (G_0) and gray value from wax paper with glucose (G).

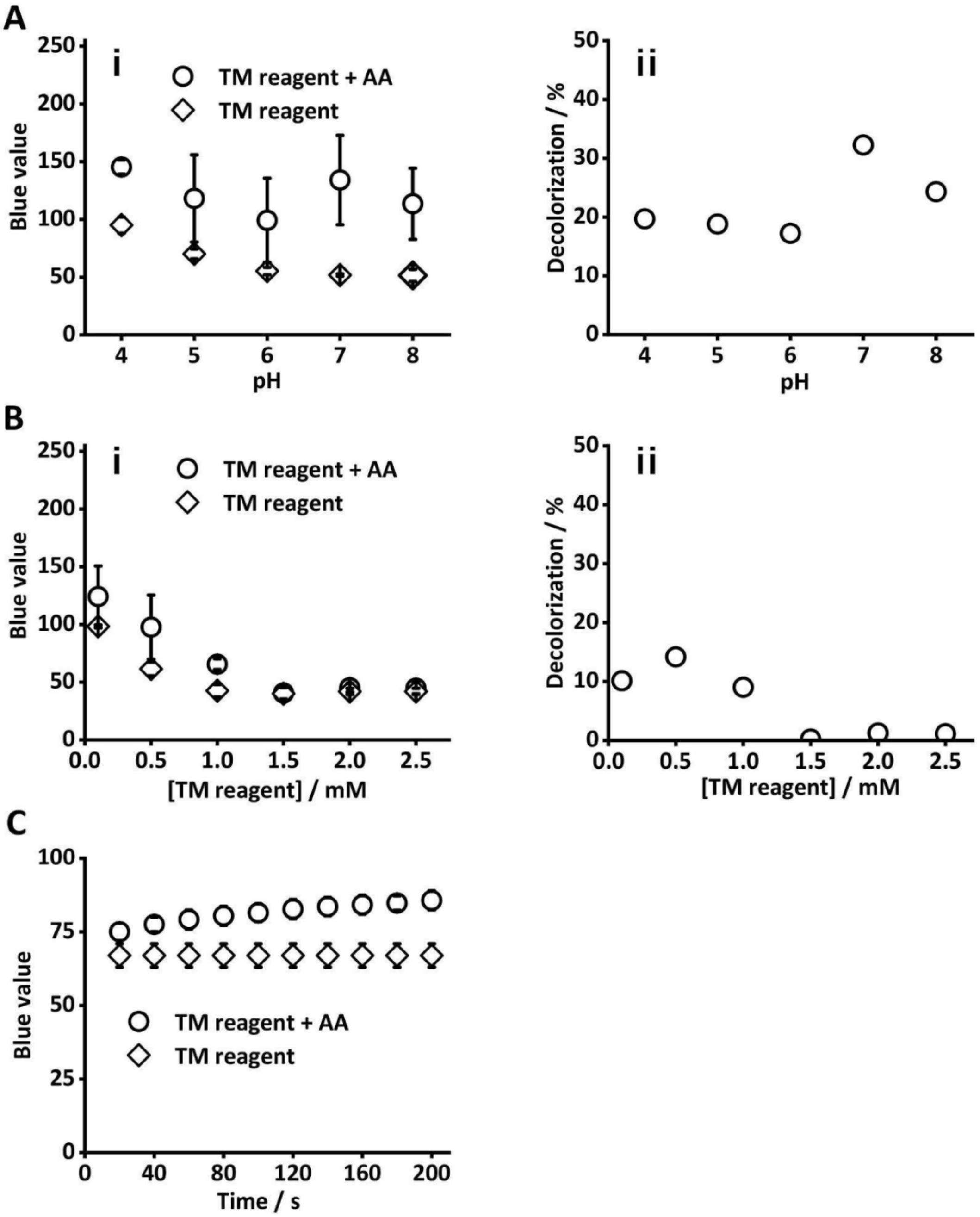

Figure S19. Optimization of AA sensing: (A) pH optimization: (i) effect of pH on TM reagent with and without AA, (ii) decolorization of TM reagent with pH change; (B) TM reagent concentration optimization: (i) effect of TM reagent concentration on decolorization of TM reagent, (ii) decolorization of TM reagent; and (C) contact time. Decolorization was calculated using the formula: *decolorization* = [(*blue value of sample – blue value of blank*)/(255)]×100; where sample is pregel with TM reagent pipetted on wax paper with 10-mm drawn circle coated with AA and blank is pregel with TM reagent pipetted on wax paper with 10-mm drawn circle without AA.

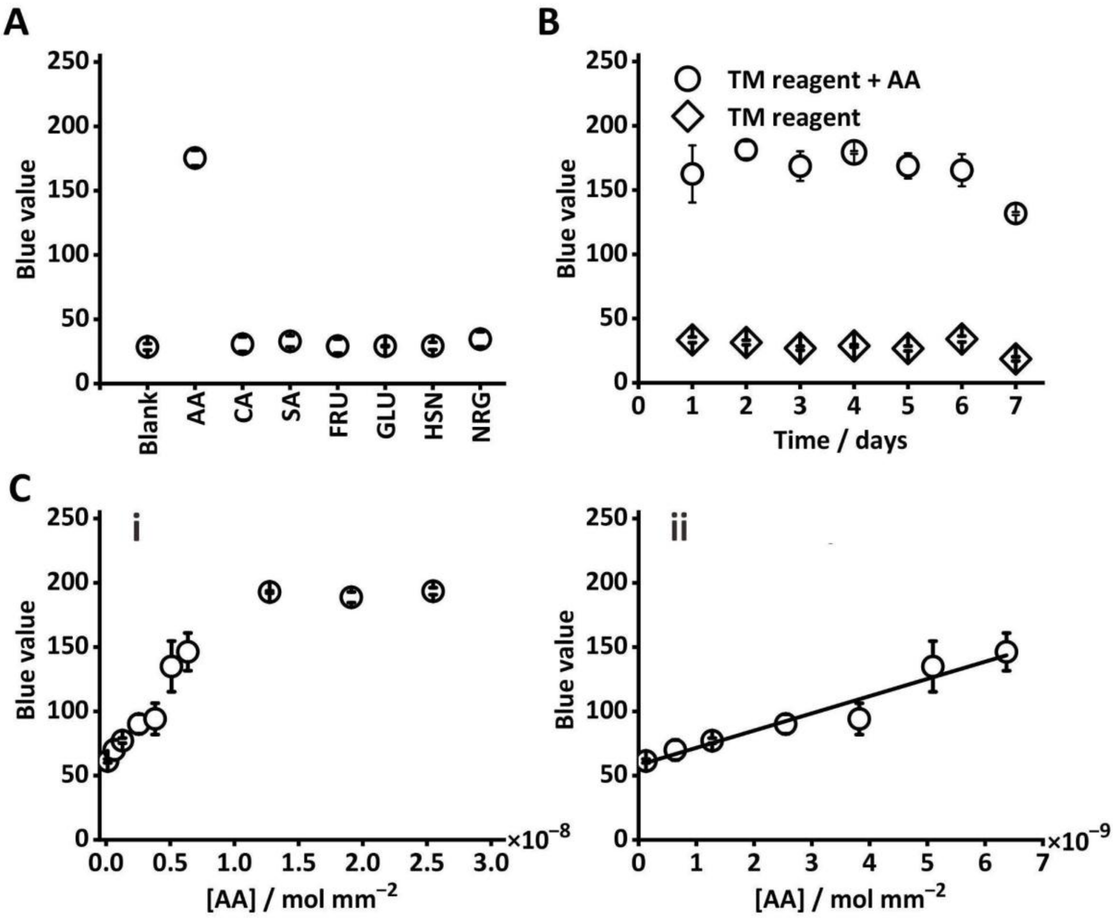

Figure S20. Characterization of sensing pregel performance for AA detection: (A) selectivity test for AA against potentially interfering analytes—caffeic acid (CA), sinapic acid (SA), FRU, GLU, hesperidin (HSN) and naringenin (NRG); (B) stability test: detection of 6.37×10^{-9} mol mm^{-2} AA on wax paper for 7 days, n = 3 each day; (C) calibration curve: (i) AA concentration at 10 levels; (ii) calibration curve from the linear response of 7 levels.

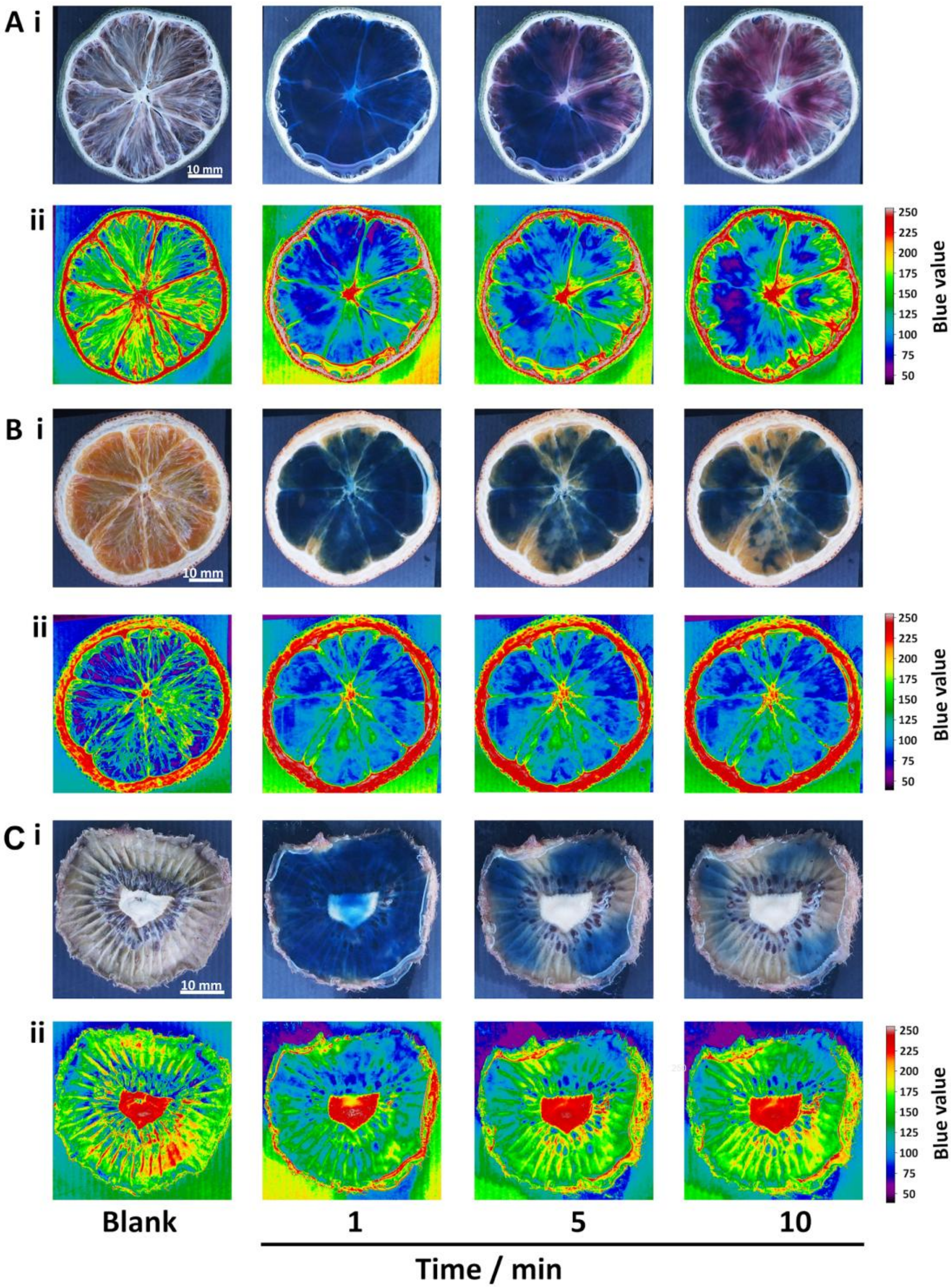

Figure S21. AA detection and mapping on sliced citrus fruit tissue: (A) lemon; (B) orange; (C) kiwi fruits. Rows (i) and (ii): (i) mixture of agarose pregel and 1 mM TM reagent applied onto sliced fruit tissue; (ii) spatial distribution of AA in sliced fruit tissue mapped as blue value intensity at different time-points. Blank: dried slice of citrus fruit tissue.

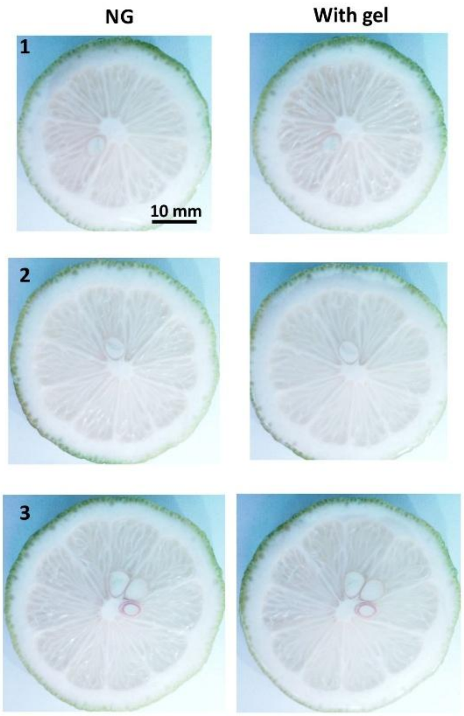

Figure S22. Comparison of lemon slice specimens without pregel (NG) and with pregel not containing TM reagent. NG: no pregel added.

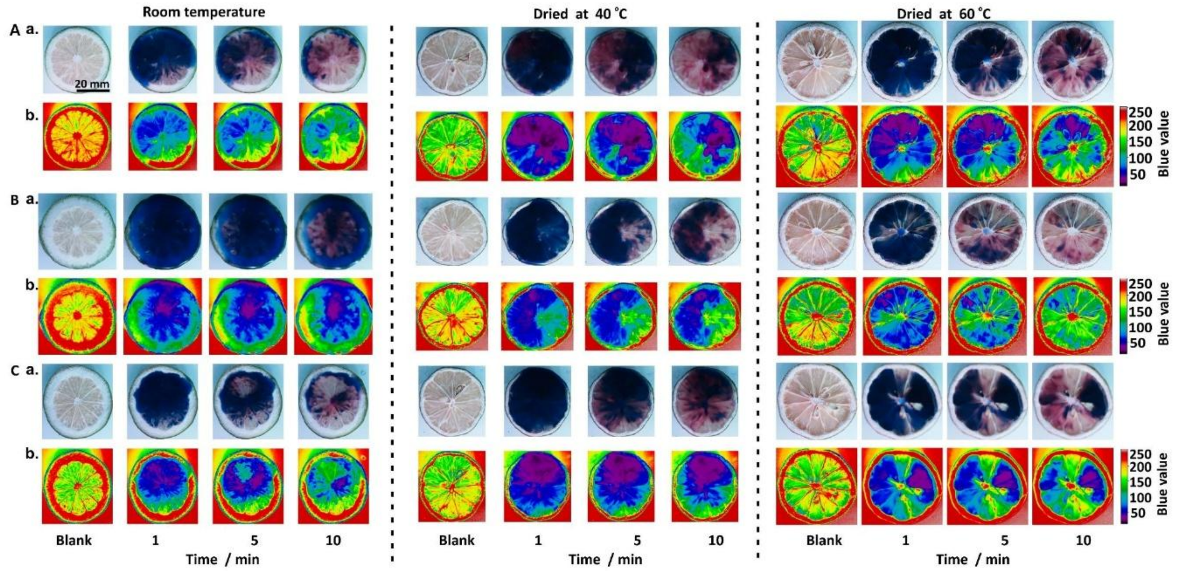

Figure S23. Comparison of AA distribution between fresh lemon fruit slices and slices dried at 40 °C and 60 °C. Three replicates (A, B, and C) are shown: (a) mixture of agarose pregel and 1 mM TM reagent applied onto sliced lemon tissue; (b) spatial distribution of AA in sliced fruit tissue mapped as blue value intensity at different time-points. Blank: dried slice of lemon fruit tissue.

ADDITIONAL REFERENCES

1. Trinder, P. Determination of Blood Glucose Using an Oxidase-Peroxidase System with a Non-Carcinogenic Chromogen. *J. Clin. Pathol.* **1969**, *22* (2), 158–161. https://doi.org/10.1136/jcp.22.2.158.
2. Marques, S. M.; Esteves Da Silva, J. C. G. Firefly Bioluminescence: A Mechanistic Approach of Luciferase Catalyzed Reactions. *IUBMB Life* **2009**, *61* (1), 6–17. https://doi.org/10.1002/iub.134.
3. Nielsen, S. S. Vitamin C Determination by Indophenol Method. In *Food Analysis Laboratory Manual*; Nielsen, S. S., Ed.; Springer International Publishing: Cham, 2017; pp 143–146. https://doi.org/10.1007/978-3-319-44127-6_15.
4. Parker, A. J.; Haskins, E. F.; Deyrup-Olsen, I. Toluidine Blue: A Simple, Effective Stain for Plant Tissues. *Am. Biol. Teach.* **1982**, *44* (8), 487–489. https://doi.org/10.2307/4447575.
5. Dwihapsari, Y.; Prabawa, N. M.; Fairuzzihab Qodarul, M. R.; Dewi, S. S.; Hajidah, D. H. The Comparison of Noninvasive Assessments of Shear Modulus Using Quantitative T2 Magnetic Resonance Imaging and Rheology of Agarose Hydrogel. *Mech. Mater.* **2022**, *171*, 104358. https://doi.org/10.1016/j.mechmat.2022.104358.
6. Ghebremedhin, M.; Seiffert, S.; Vilgis, T. A. Physics of Agarose Fluid Gels: Rheological Properties and Microstructure. *Curr. Res. Food Sci.* **2021**, *4*, 436–448. https://doi.org/10.1016/j.crfs.2021.06.003.
7. Urík, J.; Paschke, A.; Vrana, B. Diffusion Coefficients of Polar Organic Compounds in Agarose Hydrogel and Water and Their Use for Estimating Uptake in Passive Samplers. *Chemosphere* **2020**, *249*, 126183. https://doi.org/10.1016/j.chemosphere.2020.126183.
8. Adeoye, A. J.; de Alba, E. A Simple Method to Determine Diffusion Coefficients in Soft Hydrogels for Drug Delivery and Biomedical Applications. *ACS Omega* **2025**, *10*, 10852–10865. https://doi.org/10.1021/acsomega.4c06984.
9. Normand, V.; Lootens, D. L.; Amici, E.; Plucknett, K. P.; Aymard, P. New Insight into Agarose Gel Mechanical Properties. *Biomacromolecules* **2000**, *1*, 730–738. https://doi.org/10.1021/bm005583j.
10. Chauhan, N.; Narang, J.; Pundir, C. S. Fabrication of Multiwalled Carbon Nanotubes/Polyaniline Modified Au Electrode for Ascorbic Acid Determination. *Analyst* **2011**, *136* (9), 1938–1945. https://doi.org/10.1039/C0AN00218F.
11. Choudhary, K.; Subbanna, B. B.; Uniyal, S.; Sharma, P. S.; Gupta, V. K.; Raghuwanshi, S. K.; Kumar, S. Optical Sensing of Vitamin C Concentrations: A Novel Approach with Etched Fiber-Based Plasmon Sensor. In *Nanoscale Imaging, Sensing, and Actuation for Biomedical Applications XXI*; SPIE, 2024; Vol. 12858, p 1285802. https://doi.org/10.1117/12.2691324.
12. Li, R.; Liu, Z.; Xiong, Y.; Zhang, X.; Chen, L.; Li, D.; Huang, C.; Yu, S.; Jia, X. A Smartphone-Enabled Colorimetric Microneedle Sensing Platform for Rapid Detection of Ascorbic Acid in Fruits. *ACS Appl. Mater. Interfaces* **2024**, *16* (46), 63941–63950. https://doi.org/10.1021/acsami.4c15637.

13. Paixão, T. R. L. C.; Lowinsohn, D.; Bertotti, M. Use of an Electrochemically Etched Platinum Microelectrode for Ascorbic Acid Mapping in Oranges. *J. Agric. Food Chem.* **2006**, *54* (8), 3072–3077. https://doi.org/10.1021/jf052874g.

COMPUTER CODES

Python code for measuring gray value

```
import os
import cv2
import numpy as np
import pandas as pd
def get_gray_values(image_path):
    image = cv2.imread(image_path)
    gray_image = cv2.cvtColor(image, cv2.COLOR_BGR2GRAY)
         gray_values = gray_image.flatten()
    return gray_values
all_file_names = []  # Names of files read
all_gray_average_values = []  # Average gray scale value of the
files
path ="image path"
for dir,subdire, files in os.walk(path):
    # print("+++++++++++++++++++++Folder +++++++++++",dir)
    # for sub in subdire: # List of sub folders in the current
folder
       # print("folder  - ",sub)
    for file in files: # List of files in the folder
        full_path  = os.path.join(dir , file)
        # print(full_path)  # Prints full path for the file
        gray_values = get_gray_values(full_path)
        mean_gray = np.mean(gray_values)
        all_file_names.append(file)
        all_gray_average_values.append(mean_gray)
# for a in range(len(all_file_names)):
    #    print("File    ",all_file_names[a],"     =     "     ,
all_gray_average_values[a])
d = [all_file_names ,all_gray_average_values]
df = pd.DataFrame(d)
df.to_csv('file.csv',index_label="file name",index=True)
```

Python code for measuring RGB

```
import os
import cv2
import numpy as np
import pandas as pd
def get_rgb_values(image_path):
    image = cv2.imread(image_path)
```

```
    rgb_image = cv2.cvtColor(image, cv2.COLOR_BGR2RGB)
    r_values = rgb_image[0].flatten()
    g_values = rgb_image[1].flatten()
    b_values = rgb_image[2].flatten()
    return r_values, g_values, b_values
all_file_names = []  # Names of files read
all_r_average_values = []  # Average red scale value of the files
all_g_average_values = []   # Average green scale value of the
files
all_b_average_values = []   # Average blue scale value of the
files
path ="image path"
for dir ,subdire , files in os.walk(path):
    # print("+++++++++++++++++++++Folder +++++++++++",dir)
    # for sub in subdire: #List  of sub folders in the current
folder
       # print("folder  - ",sub)
    for file in files: # List of files in the folder
        full_path  = os.path.join(dir , file)
        # print(full_path)  #Print full path for the file
        r, g, b = get_gray_values(full_path)
        mean_r = np.mean(r)
        mean_g = np.mean(g)
        mean_b = np.mean(b)

        all_file_names.append(file)
        all_r_average_values.append(mean_r)
        all_g_average_values.append(mean_g)
        all_b_average_values.append(mean_b)
# for a in range(len(all_file_names)):
#      print("File      ",all_file_names[a],"       =       "
,all_r_average_values[a],
all_g_average_values[a],all_b_average_values[a])
d       =        [all_file_names        ,all_r_average_values,
all_g_average_values, all_b_average_values]
df = pd.DataFrame(d)
df.to_csv('file.csv',index_label="file name",index=True)
```

Python code for glucose mapping

```
import cv2
import matplotlib.pyplot as plt
import numpy as np
```

```
# Load image (grayscale)
image_path = r"image path.tif"
img = cv2.imread(image_path, cv2.IMREAD_GRAYSCALE)

if img is None:
    raise ValueError("Image not found. Check the file path!")

# Parameters
sizeX = m   # Number of slices along size x=size y
c = n     # Size of each slice in pixels

# Create an empty array to store mean gray values
gray_map = np.zeros((sizeX, sizeX))

# Loop through each slice and compute mean gray value
for i in range(sizeX):
    for j in range(sizeX):
        slice = img[i*c:(i+1)*c, j*c:(j+1)*c]
        mean_val = np.mean(slice)
        gray_map[i, j] = mean_val

# Plot with colorbar showing full intensity range
im   =   plt.imshow(gray_map,   cmap='nipy_spectral',   vmin=0,
vmax=255)  # Ensure consistent scaling
cbar = plt.colorbar(im)
cbar.set_label("Gray    Intensity    (0-255)",    rotation=90,
labelpad=20)
plt.title("Gray Value Map")
plt.axis("off")
plt.savefig(r"image path.PNG", dpi=300)
plt.show()
```

Python code for ATP mapping

```
import cv2
import matplotlib.pyplot as plt
import numpy as np
# Load image
img = cv2.imread("image path")
# Split channels
b, g, r = cv2.split(img)
sizeX = 25
rgbarray = np.zeros((sizeX * sizeX,3))
m = 0  # y axis
```

```
n = 0  # x axis
c = 8 # Size of a single slice
for i in range(0,sizeX):
    for j in range(0,sizeX):
        slice = img[i*c:(i+1)*c, j*c:(j+1)*c]
        ij = np.mean(slice, axis = 0)
        ij = np.mean(ij, axis = 0)
        np.set_printoptions(precision = 3)
# Show green channel as map
plt.imshow(g)
plt.title("Green Channel Mapping")
plt.axis("off")
# Create heatmap using nipy_spectral
plt.imshow(g, cmap="nipy_spectral")
plt.colorbar(label="Intensity")
plt.title("Green Channel Heatmap (Viridis)")
plt.axis("off")
# Save the heatmap
plt.savefig(r'image path.PNG', dpi=300)
plt.show()
```

Python code for ascorbic acid mapping

```
import cv2
import matplotlib.pyplot as plt
import numpy as np

# Load image
img = cv2.imread("image path")
# Split channels
b, g, r = cv2.split(img)
sizeX = 50
rgbarray = np.zeros((sizeX * sizeX,3))
m = 0  # y axis
n = 0  # x axis
c = 8 # Size of a single slice
for i in range(0,sizeX):
    for j in range(0,sizeX):
        slice = img[i*c:(i+1)*c, j*c:(j+1)*c]
        ij = np.mean(slice, axis = 0)
        ij = np.mean(ij, axis = 0)
        np.set_printoptions(precision = 3)

# Show blue channel as map
```

```
plt.imshow(g)
plt.title("Blue Channel Mapping")
plt.axis("off")
# Create heatmap using nipy_spectral
plt.imshow(b, cmap="nipy_spectral")
plt.colorbar(label="Intensity")
plt.title("Blue Channel Heatmap (Viridis)")
plt.axis("off")
# Save the heatmap
plt.savefig(r'image path.PNG', dpi=300)
plt.show()
```
